# The Clinical Significance of O^6^-Methylguanine-DNA Methyltransferase Promoter Methylation Status in Adult Patients With Glioblastoma: A Meta-analysis

**DOI:** 10.3389/fneur.2018.00127

**Published:** 2018-03-21

**Authors:** Yu-Hang Zhao, Ze-Fen Wang, Chang-Jun Cao, Hong Weng, Cheng-Shi Xu, Kai Li, Jie-Li Li, Jing Lan, Xian-Tao Zeng, Zhi-Qiang Li

**Affiliations:** ^1^Department of Neurosurgery, Zhongnan Hospital, Wuhan University, Wuhan, China; ^2^Department of Physiology, School of Basic Medical Sciences, Wuhan University, Wuhan, China; ^3^Center for Evidence-Based and Translational Medicine, Zhongnan Hospital, Wuhan University, Wuhan, China

**Keywords:** O^6^-methylguanine-DNA methyltransferase, methylation, glioblastoma, prognosis, temozolomide

## Abstract

**Background and objective:**

Promoter status of O^6^-methylguanine-DNA methyltransferase (*MGMT*) has been widely established as a clinically relevant factor in glioblastoma (GBM) patients. However, in addition to varied therapy schedule, the prognosis of GBM patients is also affected by variations of age, race, primary or recurrent tumor. This study comprehensively investigated the association between *MGMT* promoter status and prognosis in overall GBM patients and in different GBM subtype including new diagnosed patients, recurrent patients and elderly patients.

**Methods:**

A comprehensive search was performed using PubMed, EMBASE, Cochrane databases to identify literatures (published from January 1, 2005 to April 1, 2017) that evaluated the associations between *MGMT* promoter methylation and prognosis of GBM patients.

**Results:**

Totally, 66 studies including 7,886 patients met the inclusion criteria. Overall GBM patients with a methylated status of *MGMT* receiving temozolomide (TMZ)-containing treatment had better overall survival (OS) and progression-free survival (PFS) [OS: hazard ratio (HR) = 0.46, 95% confidence interval (CI): 0.41–0.52, *p* < 0.001, Bon = 0.017; PFS: HR = 0.48, 95% CI 0.40–0.57, *p* < 0.001, Bon = 0.014], but no significant advantage on OS or PFS in GBM patients with TMZ-free treatment was observed (OS: HR = 0.97, 95% CI 0.91–1.03, *p* = 0.08, Bon = 1; PFS: HR = 0.76, 95% CI 0.57–1.02, *p* = 0.068, Bon = 0.748). These different impacts of MGMT status on OS were similar in newly diagnosed GBM patients, elderly GBM patients and recurrent GBM. Among patients receiving TMZ-free treatment, survival benefit in Asian patients was not observed anymore after Bonferroni correction (Asian OS: HR = 0.78, 95% CI 0.64–0.95, *p* = 0.02, Bon = 0.24, *I*^2^ = 0%; PFS: HR = 0.69, 95% CI 0.50–0.94, *p* = 0.02, Bon = 0.24). No benefit was observed in Caucasian receiving TMZ-free therapy regardless of Bonferroni adjustment.

**Conclusion:**

The meta-analysis highlights the universal predictive value of *MGMT* methylation in newly diagnosed GBM patients, elderly GBM patients and recurrent GBM patients. For elderly methylated GBM patients, TMZ alone therapy might be a more suitable option than radiotherapy alone therapy. Future clinical trials should be designed in order to optimize therapeutics in different GBM subpopulation.

## Introduction

Glioblastoma (GBM) is the most frequent primary malignant brain tumor with poor prognosis. From 2005, radiotherapy combined with concomitant and adjuvant temozolomide (TMZ) after surgical maximal safe resection, namely STUPP treatment, has been widely used for newly diagnosed GBM patients less than 65 years old (1, 2). A phase III trial showed that tumor treatment fields, a novel cancer treatment modality, had similar efficacy as chemotherapy regimens in recurrent GBM (3). However, limited improvement of the overall survival (OS) has been achieved in patients with GBM (4, 5). Therefore, identification of biomarkers determining tumor response to treatment may help in developing targeted therapy or optimize patients’ management.

O-6-methylguanine-DNA methyltransferase (MGMT) is a ubiquitously expressed DNA repair enzyme. MGMT protein removes alkyl adducts at the O^6^ position of guanine, thereby neutralizing the cytotoxic effects of alkylating agents such as TMZ (6, 7). High MGMT expression in glioma cells is the predominant mechanism underlying tumor resistance to alkylating agents (8–10). Meanwhile, status of *MGMT* promoter methylation is associated with tumor response to TMZ therapy (11, 12). *MGMT* promoter methylation, resulting in transcriptional silencing, correlates well with improved survival in GBM patients exposed to alkylating agents’ treatment (13–15). Results of European Organization for Research and Treatment of Cancer and National Cancer Institute of Canada trial indicated that *MGMT* promoter methylation was the strongest predictor for outcome and benefit from TMZ (2, 16). Accordingly, this biomarker is currently used for clinical decision-making and stratifying or selecting GBM patients for clinical trials (17).

Although *MGMT* promoter methylation has a strong influence on response to TMZ and clinical outcome in GBM patients, its prognostic value on GBM patients remains ambiguous. Some studies indicated that it was associated with better outcome in methylated patients receiving TMZ-containing therapy (18, 19). But some studies also showed that it conferred survival benefit in methylated patients receiving TMZ-free therapy (21, 22). So it is necessary to review whether the survival benefit from MGMT methylation is therapy dependent or independent, which will define MGMT promoter methylation as a predictive or prognostic biomarker. In addition to varied therapy schedules, the outcome and survival of GBM patients may be affected by other prognostic variables, including primary or recurrent tumor, age and race. Thus, we conducted a comprehensive and exact analysis on the association between *MGMT* promoter methylation and prognosis in overall GBM patients as well as in different GBM subpopulation, including newly diagnosed patients, recurrent patients, elderly patients and patients with different races. This meta-analysis will provide an updated and precise review on the clinical value of *MGMT* promoter methylation on progression-free survival (PFS) and OS in GBM patients.

## Methods

### Search Strategy

We performed a systematic review to identify all related articles from PubMed, EMBASE and the Cochrane Library covering the association of *MGMT* methylation with prognosis and data of hazard ratios (HRs) and 95% confidence intervals (CIs). The articles enrolled in analysis were published between January 1, 2005 and April 1, 2017. The following subject terms were used: (1) “Glioblastoma,” “GBM,” “High-Grade Glioma,” “Astrocytoma, Grade IV,” “Astrocytomas, Grade IV,” “Glioblastoma Multiform,” or “Glioblastomas”; (2) “MGMT” or “O-6-methylguanine-DNA methyltransferase.” The eligible studies were restricted to human beings.

### Inclusion and Exclusion Criteria

We evaluated the eligible studies only if all the following conditions were met: (1) studies investigated the relation between *MGMT* promoter methylation and survival in GBM patients; (2) treatment schedules and testing methods were all included; (3) HR and 95% CI for OS and PFS were available directly or calculated using the Kaplan–Meier survival curves; and (4) specific drugs for chemotherapy were introduced.

### Study Selection and Data Extraction

Study selection was independently performed by two authors and disagreements were resolved through discussion. The following data were extracted: the author’s name, country, publication year, number of patients, treatment detail, outcomes (including HRs and 95% CIs), the Cox regression model, and study design feature.

### Quality Assessment

The bias risk in each study was independently assessed by two authors using a modified domain-based Newcastle-Ottawa Scale (NOS) for non-randomized studies. The assessment included selection bias, performance bias, detection bias, attrition bias and reporting bias. Important prognostic variables, including age, neurologic status, extent of resection, tumor location, primary or recurrent GBM and *MGMT* promoter status, were added into NOS according to the Reporting Recommendations for Tumor Marker Prognostic Studies (REMARK) checklist for a tumor prognostic study (23, 24). The judgment criteria for the modified evaluation were explicitly described in Table S1 in Supplementary Material.

### Statistical Analysis

The statistical analysis was performed by STATA 12.0 software. HR and 95% CI were directly extracted or calculated using the Kaplan–Meier survival curves or the methods reported by Tierney et al. (25). To evaluate the association of *MGMT* promoter methylation with OS and PFS, pooled HRs of methylated GBM patients were compared to those of unmethylated patients. Subgroup analysis was performed to evaluate whether methylated patients benefit from different therapies (TMZ-containing, TMZ-free alkylating agents, or radiotherapy alone). The statistical heterogeneity among studies was assessed by *Q*-test and *I*^2^ statistics (26). If there was no obvious heterogeneity, fixed-effect model was used to estimate the pooled HR (27); otherwise, random-effect model was used (28). Bonferroni method was used for multiple comparison adjustments. Publication bias was assessed by funnel plots and Egger’s test (29), and a trim and fill method was applied to estimate asymmetry in funnel plots (30). Sensitivity analysis by deleting each enrolled study in turn was conducted to assess overall robustness of the meta-analysis results.

## Results

### Characteristics of Studies

**Figure 1:**
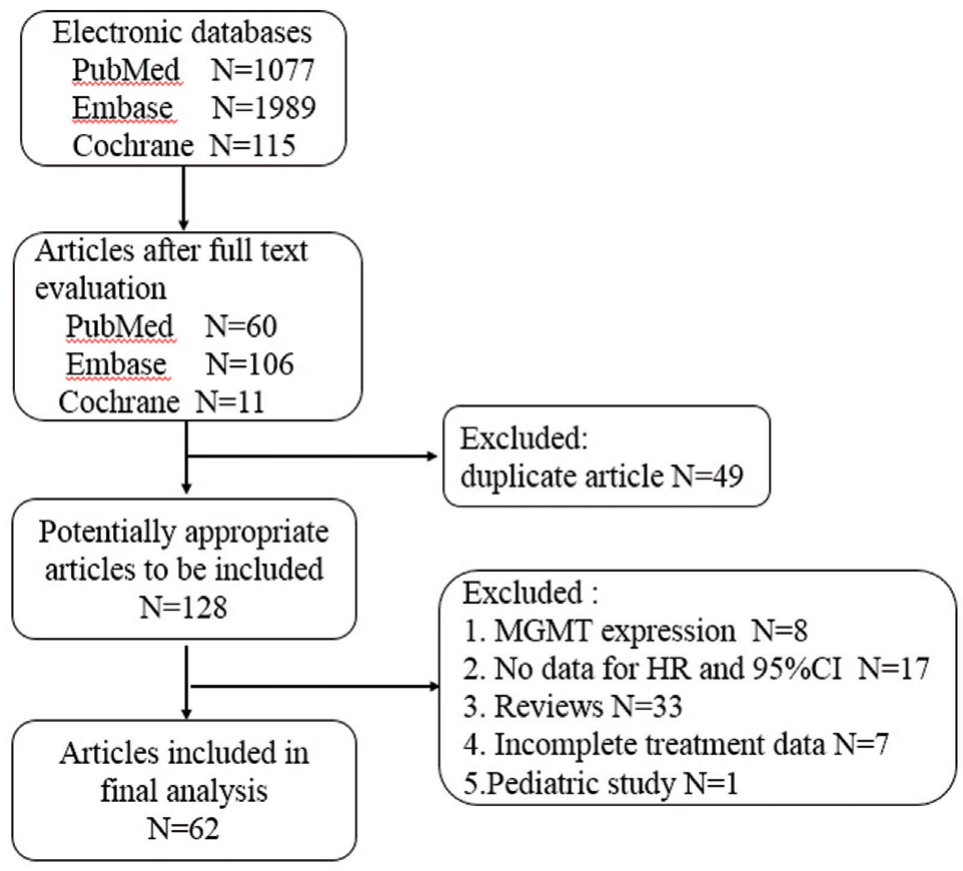
Flow diagram of study selection.

The flow chart of literature selection was presented in Figure 1. Totally, 3,181 articles were screened. Finally, a total of 7,886 patients in 66 studies (four articles comprising two individual trials were extracted as eight individual studies) were identified, including 7 randomized trials, 59 non-randomized trials. Of these 66 studies, 54 studies were related to TMZ-containing chemotherapy and 12 studies were related to TMZ-free treatment (4 studies of radiotherapy alone and 12 studies of TMZ-free alkylating agents chemotherapy). The characteristics of all studies are summarized in Table 1. Quality assessment showed no apparent variations among the studies in most domains of bias except for selection bias (see Table S1 in Supplementary Material).

**Table 1:** Characteristics of included studies.

Author	Country	Study type	Cox	Patients (*N*)	OS HR (95% CI)	Type of cancer	Treatment after resection	Race	Methylation assay method
Arita et al. (31)	Japan	Retrospective	Multivariate	453	0.43 (0.33, 0.56)	GBM	RT + TMZ	Asian	Pyrosequencing
Arvold et al. (32)	America	Non-RCT	Univariate	55	0.47 (0.27, 0.81)	GBM	RT + TMZ	Mixed race	NA
Azoulay et al. (33)	Canada	Non-RCT	Multivariate	276	0.46 (0.33, 0.64)	GBM	RT + TMZ	Caucasian	NA
Brandes et al. (34)	Italy	Non-RCT	Multivariate	119	0.66 (0.47, 0.94)	GBM	RT + TMZ	Caucasian	MSP
Brandes et al. (22)	Italy	Non-RCT	Univariate	25	0.19 (0.04, 0.99)	Recurrent GBM	RT + FTM	Caucasian	MSP
Chen et al. (35)	China	Non-RCT	Multivariate	128	0.65 (0.41, 1.01)	GBM	RT	Asian	NA
Clarke et al. (36)	America	RCT	Univariate	85	0.42 (0.13, 1.39)	GBM	RT + TMZ	Mixed race	MSP
Cominelli et al. (37)	Italy	Non-RCT	Univariate	70	0.12 (0.01, 0.98)	GBM	RT + TMZ	Caucasian	MSP
Etcheverry et al. (38)	Spain	Non-RCT	Multivariate	399	0.33 (0.24, 0.46)	GBM	RT + TMZ	Caucasian	MSP and Pyrosequencing
Gallego Perez-Larraya et al. (39)	France	Non-RCT	Multivariate	31	0.43 (0.20, 0.93)	GBM	TMZ	Caucasian	MSP
Gilbert et al. (40)	America	RCT	Univariate	760	0.58 (0.48, 0.69)	GBM	RT + TMZ	Mixed race	MSP
Giordano et al. (41)	Germany	Non-RCT	Univariate	65	1.31 (0.75, 2.28)	GBM	RT + TMZ + Celecoxid	Caucasian	NA
Glas et al. (42)	Switzerland	Non-RCT	Univariate	23	0.43 (0.22, 0.76)	GBM	RT + TMZ + CCNU	Caucasian	MSP
Grossman et al. (43)	America	Non-RCT	Multivariate	122	0.85 (0.56, 1.31)	GBM	RT + TMZ + BCNU	Mixed race	MSP
Gutenberg et al. (44)	Germany	Non-RCT	Univariate	17	0.62 (0.43, 0.90)	Recurrent GBM	BCNU + TMZ	Caucasian	MSP
Gutenberg et al. (44)	Germany	Non-RCT	Univariate	13	0.99 (0.94, 1.04)	GBM	BCNU	Caucasian	MSP
Han et al. (45)	China	Non-RCT	Multivariate	152	0.66 (0.44, 0.98)	GBM	RT + TMZ	Asian	MSP
Jungk et al. (46)	Germany	Non-RCT	Multivariate	63	0.89 (0.51, 1.53)	Recurrent GBM	RT + BCNU	Caucasian	MSP
Kerkhof et al. (47)	France	Non-RCT	Multivariate	47	1.04 (0.84, 1.29)	GBM	RT + TMZ	Caucasian	NA
Kim et al. (48)	Korea	Non-RCT	Multivariate	70	0.30 (0.14, 0.65)	GBM	RT + TMZ	Asian	NA
Kim et al. (49)	Korea	Non-RCT	Multivariate	78	0.56 (0.40, 0.83)	GBM	RT + TMZ	Asian	MSP
Kreth et al. (50)	Germany	Non-RCT	Multivariate	222	0.30 (0.22, 0.41)	GBM	RT + TMZ	Caucasian	MSP
Lai et al. (51)	America	Non-RCT	Multivariate	70	0.49 (0.34, 0.71)	GBM	RT + TMZ + BEV	Mixed race	MSP
Lakomy et al. (52)	Czech Republic	Non-RCT	Univariate	38	0.40 (0.21, 0.78)	GBM	RT + TMZ	Caucasian	MS-HRM
Lam and Chambers (53)	Canada	Non-RCT	Univariate	101	0.64 (0.38, 1.08)	GBM	RT + TMZ	Caucasian	MSP
Lee et al. (54)	Korea	Non-RCT	Multivariate	36	0.22 (0.04, 1.12)	GBM	RT + TMZ	Asian	MSP
Liu et al. (21)	China	Non-RCT	Multivariate	137	0.88 (0.58, 1.26)	Recurrent GBM	BEV + FTM	Asian	MSP
Lombardi et al. (55)	Italy	Non-RCT	Multivariate	151	0.2 (0.10, 0.50)	GBM	RT + TMZ	Caucasian	MSP
Lombardi et al. (56)	Italy	Non-RCT	Univariate	34	0.80 (0.65, 0.97)	Recurrent GBM	TMZ + FTM	Caucasian	MSP
Ma et al. (57)	China	Non-RCT	Multivariate	56	0.44 (0.19, 0.83)	GBM	RT + TMZ + ELE	Asian	MSP
Malmström et al. (58)	Europe (multicenter)	RCT	Univariate	72	0.56 (0.34, 0.93)	GBM	TMZ	Caucasian	MSP
Malmström et al. (58)	Europe (multicenter)	RCT	Univariate	131	0.97 (0.69, 1.38)	GBM	RT	Caucasian	MSP
Metellus et al. (59)	France	Non-RCT	Multivariate	61	0.10 (0.02, 0.37)	GBM	RT + TMZ	Caucasian	MSP
Metellus et al. (60)	France	Non-RCT	Multivariate	21	0.19 (0.06, 0.77)	Recurrent GBM	TMZ + BCNU	Caucasian	MSP
Minniti et al. (61)	Italy	Non-RCT	Multivariate	243	0.30 (0.21, 0.42)	GBM	RT + TMZ	Caucasian	MSP
Minniti et al. (62)	Italy	Non-RCT	Multivariate	83	0.41 (0.22, 0.75)	GBM	RT + TMZ	Caucasian	MSP
Minniti et al. (63)	Italy	Non-RCT	Multivariate	36	0.40 (0.19, 0.94)	Recurrent GBM	RT + TMZ	Caucasian	MSP
Montano et al. (64)	Italy	Non-RCT	Multivariate	73	0.72 (0.37, 1.37)	GBM	RT + TMZ	Caucasian	MSP
Motomura et al. (65)	Japan	Non-RCT	Multivariate	68	0.38 (0.18, 0.83)	GBM	RT + TMZ + β-IFN	Asian	Pyrosequencing
Murat et al. (66)	Germany	Non-RCT	Multivariate	42	0.06 (0.001, 0.20)	GBM	RT + TMZ	Caucasian	NA
Nguyen et al. (67)	America	Non-RCT	Multivariate	303	0.39 (0.30, 0.52)	GBM	RT + TMZ + BEV	Mixed race	MSP
Niyazi et al. (68)	Germany	Non-RCT	Univariate	30	0.28 (0.10, 0.77)	GBM	RT + TMZ	Caucasian	MSP
Park et al. (69)	Korea	Non-RCT	Multivariate	48	0.81 (0.43, 1.52)	GBM	RT + ACNU + CDDP	Asian	MSP
Perry et al. (70)	Canada and Europe	RCT	Univariate	281	0.93 (0.68, 1.21)	GBM	RT	Caucasian	MSP
Rosati et al. (71)	Italy	Non-RCT	Multivariate	47	0.27 (0.12, 0.60)	GBM	RT + TMZ	Caucasian	MSP
Sana et al. (72)	Czech Republic	Non-RCT	Univariate	58	0.51 (0.29, 0.91)	GBM	RT + TMZ	Caucasian	MS-HRM
Saraiva-Esperon et al. (73)	America	Non-RCT	Multivariate	159	0.52 (0.36, 0.73)	GBM	RT + TMZ	Caucasian	MSP
Saraiva-Esperon et al. (73)	Australia	Non-RCT	Multivariate	144	0.42 (0.28, 0.63)	GBM	RT + TMZ	Mixed race	Pyrosequencing
Schaich et al. (74)	Germany	Non-RCT	Multivariate	61	0.88 (0.36, 2.15)	GBM	RT + TMZ	Caucasian	MSP
Schaub et al. (75)	Germany	Non-RCT	Univariate	143	1.13 (0.77, 1.66)	Recurrent GBM	RT + BEV + CPT-11	Caucasian	NA
Shenouda et al. (76)	Canada	Non-RCT	Univariate	48	0.40 (0.19, 0.77)	GBM	RT + TMZ	Caucasian	NA
Soffietti et al. (77)	Italy	Non-RCT	Multivariate	38	0.82 (0.38, 1.74)	Recurrent GBM	BEV + FTM	Caucasian	MSP
Stummer et al. (78)	Germany	Non-RCT	Univariate	79	0.23 (0.10, 0.52)	GBM	RT + TMZ	Caucasian	MSP
Stupp et al. (79)	Europe(multicenter)	Non-RCT	Univariate	55	0.44 (0.21, 0.91)	GBM	RT + TMZ + Cilengitide	Caucasian	MSP
Thon et al. (80)	Germany	Non-RCT	Multivariate	56	0.31 (0.16, 0.58)	GBM	RT + TMZ (unresectable)	Caucasian	MSP
Vaios et al. (81)	America	Non-RCT	Multivariate	86	0.11 (0.04, 0.26)	GBM	TMZ	Mixed race	NA
Van Mieghem et al. (82)	Belgium	Non-RCT	Multivariate	112	0.70 (0.27, 1.8)	GBM	RT + TMZ	Caucasian	MSP
Wee et al. (83)	Korea	Non-RCT	Multivariate	340	0.54 (0.41, 0.70)	GBM	RT + TMZ	Asian	MSP
Weller et al. (19)	Europe(multicenter)	Non-RCT	Univariate	105	0.55 (0.44, 0.68)	Recurrent GBM	RT + TMZ	Caucasian	MSP
Wick et al. (84)	Europe(multicenter)	RCT	Univariate	101	0.96 (0.56, 1.63)	GBM	RT	Caucasian	MSP
Wick et al. (84)	Europe(multicenter)	RCT	Univariate	108	0.44 (0.27, 0.72)	GBM	TMZ	Caucasian	MSP
Yang et al. (85)	China	Non-RCT	Multivariate	206	0.78 (0.57, 1.04)	GBM	RT + BCNU	Asian	MSP
Yang et al. (86)	China	Non-RCT	Multivariate	238	0.59 (0.37, 0.95)	GBM	RT + TMZ	Asian	Pyrosequencing
Zhang et al. (87)	China	Non-RCT	Multivariate	154	0.24 (0.15, 0.39)	GBM	RT + TMZ	Asian	NA

**Author**	**Country**	**Study type**	**Cox**	**Patients (***N***)**	**OS HR (95% CI)**	**Type of cancer**	**Treatment after resection**	**Race**	**Testing methods**

Lai et al. (51)	America	Non-RCT	Multivariate	70	0.47 (0.32, 0.70)	GBM	RT + TMZ + BEV	Mixed race	MSP
Shenouda et al. (76)	Canada	Non-RCT	Univariate	48	0.47 (0.22, 0.78)	GBM	RT + TMZ	Caucasian	NA
Soffietti et al. (77)	Italy	Non-RCT	Multivariate	38	0.48 (0.21, 1.09)	Recurrent GBM	BEV + FTM	Caucasian	MSP
Stupp et al. (79)	Europe (multicenter)	Non-RCT	Univariate	45	0.26 (0.13, 0.51)	GBM	RT + TMZ + Cilengitide	Caucasian	MSP
Arita et al. (31)	Japan	Non-RCT	Multivariate	453	0.48 (0.37, 0.61)	GBM	RT + TMZ	Asian	Pyrosequencing
Lee et al. (54)	Korea	Non-RCT	Multivariate	36	0.40 (0.15, 1.1)	GBM	RT + TMZ	Asian	MSP
Metellus et al. (59)	France	Non-RCT	Multivariate	61	0.42 (0.21, 0.92)	GBM	RT + TMZ	Caucasian	MSP
Metellus et al. (60)	France	Non-RCT	Multivariate	21	0.15 (0.08, 0.48)	Recurrent GBM	TMZ + BCNU	Caucasian	MSP
Minniti et al. (61)	Italy	Non-RCT	Multivariate	243	0.29 (0.21, 0.40)	GBM	RT + TMZ	Caucasian	MSP
Minniti et al. (63)	Italy	Non-RCT	Multivariate	36	0.38 (0.18, 0.79)	Recurrent GBM	RT + TMZ	Caucasian	MSP
Ohno et al. (88)	Japan	Non-RCT	Multivariate	88	0.35 (0.21, 0.59)	GBM	RT + TMZ + ACNU	Asian	Pyrosequencing
Thon et al. (80)	Germany	Non-RCT	Multivariate	56	0.32 (0.17, 0.59)	GBM	RT + TMZ	Caucasian	MSP
Weller et al. (19)	Europe (multicenter)	Non-RCT	Univariate	105	0.57 (0.35, 0.90)	Recurrent GBM	RT + TMZ	Caucasian	MSP
Gilbert et al. (40)	America	RCT	Univariate	760	0.61 (0.52, 0.73)	GBM	RT + TMZ	Mixed race	MSP
Cominelli et al. (37)	Italy	Non-RCT	Univariate	70	0.29 (0.04, 2.24)	GBM	RT + TMZ	Caucasian	MSP
Giordano et al. (41)	Germany	Non-RCT	Univariate	65	2.04 (1.04, 4.00)	GBM	RT + TMZ	Caucasian	NA
Gutenberg et al. (44)	Germany	Non-RCT	Univariate	13	0.93 (0.70, 1.24)	GBM	BCNU	Caucasian	MSP
Gutenberg et al. (44)	Germany	Non-RCT	Univariate	17	0.60 (0.33, 1.07)	Recurrent GBM	BCNU + TMZ	Caucasian	MSP
Kim et al. (89)	Korea	Non-RCT	Multivariate	72	0.47 (0.27, 0.82)	Recurrent GBM	RT + TMZ	Asian	MSP
Kim et al. (49)	Korea	Non-RCT	Multivariate	78	0.63 (0.46, 0.91)	GBM	RT + TMZ	Asian	MSP
Lakomy et al. (52)	Czech Republic	Non-RCT	Univariate	38	0.48 (0.25, 0.92)	GBM	RT + TMZ	Caucasian	MS-HRM
Liu et al. (21)	China	Non-RCT	Multivariate	137	0.69 (0.52, 0.97)	Recurrent GBM	BEV + FTM	Asian	MSP
Lombardi et al. (56)	Italy	Non-RCT	Univariate	34	0.72 (0.59, 0.87)	Recurrent GBM	TMZ + FTM	Caucasian	MSP
Nguyen et al. (67)	America	Non-RCT	Multivariate	303	0.43 (0.33, 0.57)	GBM	RT + TMZ + BEV	Mixed race	MSP
Sana et al. (72)	Czech Republic	Non-RCT	Univariate	58	0.54 (0.23, 0.96)	GBM	RT + TMZ	Caucasian	MS-HRM

*Studies enrolled for OS analysis. TMZ, temozolomide; RCT, randomized control trial; RT, radiotherapy; BCNU, carmustine; FTM; fotemustine; BEV, bevacizumab; CCNU, lomustine; ELE, β-element; ACNU, nimustine; CDDP, cisplatin; β-IFN, interferon-β; CPT-11, irinotecan; MSP, methylation-specific PCR; NA, not available*.

*Studies enrolled for PFS analysis. TMZ, temozolomide; RCT, randomized control trial. RT, radiotherapy; BCNU, carmustine; FTM; fotemustine; BEV, bevacizumab; ACNU, nimustine; MSP, methylation-specific PCR; NA, not available*.

### Association between *MGMT* Promoter Methylation and Survival in Overall GBM Patients

**Figure 2:**
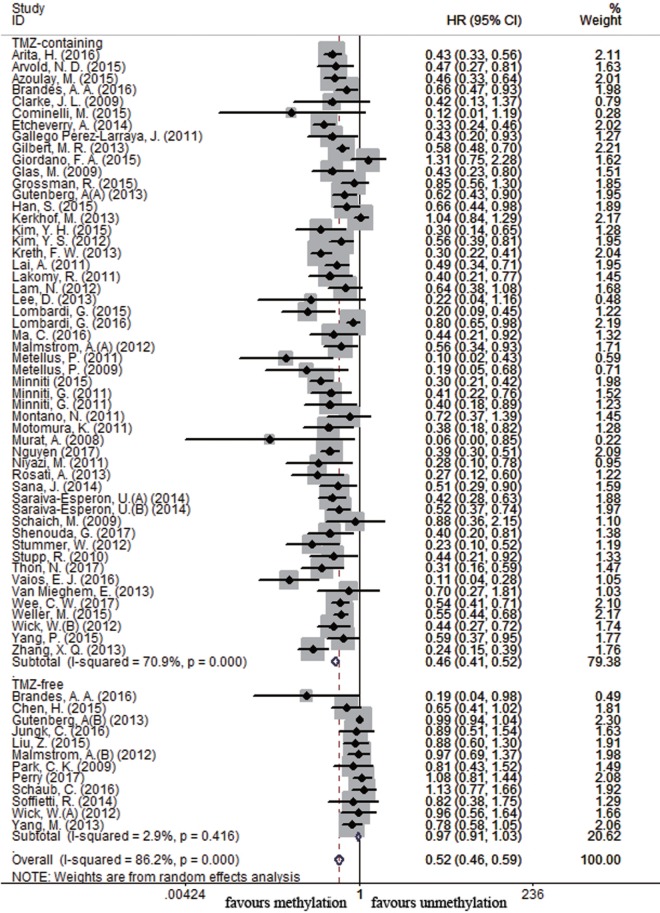
Calculated hazard ratios (HRs) and 95% confidence intervals (CIs) for the relationship between methylation and overall survival benefit from temozolomide (TMZ)-containing or TMZ-free therapy in overall glioblastoma patients (methylated vs. unmethylated patients).

**Figure 3:**
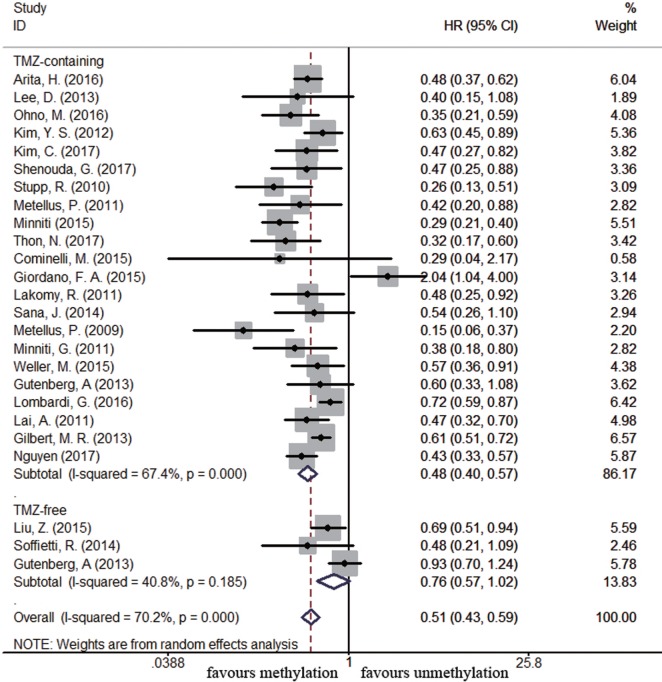
Calculated hazard ratios (HRs) and 95% confidence intervals (CIs) for the relationship between methylation and progression-free survival benefit from temozolomide (TMZ)-containing or TMZ-free therapy in overall glioblastoma patients (methylated vs. unmethylated patients).

Sixty-four and 25 studies were included to describe the correlation of *MGMT* methylation status with OS and PFS in GBM patients, respectively. GBM patients with *MGMT* promoter methylation had significantly better OS and PFS than those with unmethylated status (OS: HR = 0.52, 95% CI 0.46–0.59, *p* < 0.001, *I*^2^ = 86.2%; PFS: HR = 0.51, 95% CI 0.43–0.59, *p* < 0.001, *I*^2^ = 70.2%; see Figure S1 in Supplementary Material), indicating the association between methylation and survival benefit in GBM patients. Next, subgroup analysis was conducted to evaluate whether methylated GBM patients could benefit from different therapies. The results of subgroup analysis were summarized in Table 2. Our analysis showed that, among patients exposed to TMZ-containing treatment, methylated patients had longer OS and PFS than unmethylated patients (OS: HR = 0.46, 95% CI 0.41–0.52, *p* < 0.001, Bon = 0.017, *I*^2^ = 70.9%, Figure 2; PFS: HR = 0.48, 95% CI 0.40–0.57, *p* < 0.001, Bon = 0.014, *I*^2^ = 67.4%, Figure 3). However, no significant OS benefit from TMZ-free treatment was observed in methylated patients by analysis of 12 studies (21, 35, 44, 58, 69, 70, 77, 84, 85) (HR = 0.97, 95% CI 0.91–1.03, *p* = 0.32, *I*^2^ = 2.9%, Figure 2). Further analysis showed that methylated patients derived no OS benefit from TMZ-free alkylating agents chemotherapy (HR = 0.97, 95% CI 0.93–1.03, *p* = 0.41, Bon = 1, I2 = 9.1%). Similarly, PFS was not significantly prolonged in methylated patients with TMZ-free alkylating agents chemotherapy (HR = 0.76, 95% CI 0.57–1.02, *p* = 0.40, Bon = 0.748, *I*^2^ = 40.8%, Figure 3). These results indicate that MGMT methylation is predictive for better response to TMZ therapy in GBM patients.

**Table 2:** Summary of subgroup analysis.

Variable	Subgroup	Treatment	Trial (*N*)	HR (95% CI)	*P*-value for HR	Bon	*I^2^*	*P*-value (Egger’)
**OS analysis (methylated vs. unmethylated)**								

Overall		TMZ-containing	52	0.46 (0.41–0.52)	<0.001	0.017	70.9%	0.001
		TMZ-free	12	0.97 (0.91–1.03)	0.32	1	2.90%	0.053

Race	Caucasian	TMZ-containing	34	0.46 (0.39–0.55)	<0.001	0.017	75.5%	0.003
		TMZ-free	8	0.99 (0.94–1.04)	0.71	1	0%	0.27
	Asian	TMZ-containing	10	0.48 (0.42–0.54)	<0.001	0.017	43.8%	0.26
		TMZ-free	4	0.78 (0.64–0.95)	0.015	0.24	0%	NA
	Mixed race	TMZ-containing	8	0.48 (0.38–0.62)	<0.001	0.017	67.7%	0.302
		TMZ- free	0	NA	NA	NA	NA	NA

Study type	non-RCT	TMZ-containing	48	0.46 (0.40–0.52)	<0.001	0.017	72.9%	0.001
		TMZ-free	9	0.90 (0.78–1.03)	0.13	1	26.3%	0.033
	RCT	TMZ-containing	4	0.56 (0.48–0.65)	<0.001	0.017	0%	NA
		TMZ-free	3	1.02 (0.83–1.25)	0.83	1	0%	NA

GBM Type	Newly diagnosed	TMZ-containing	47	0.45 (0.40–0.52)	<0.001	0.017	69.80%	0.007
		TMZ-free	7	0.97 (0.90–1.04)	0.374	1	5.6%	NA
	Elderly	TMZ-containing	8	0.46 (0.32–0.65)	<0.001	0.017	71%	0.695
		TMZ-free	3	1.02 (0.83–1.25)	0.83	1	0%	NA
	Recurrent	TMZ-containing	5	0.59 (0.44–0.78)	<0.001	0.017	65%	NA
		TMZ-free	5	0.92 (0.70–1.19)	0.52	1	16.40%	NA

**PFS analysis (methylated vs. un-methylated)**								

Overall		TMZ-containing	22	0.48 (0.40–0.57)	<0.001	0.014	67.4%	0.092
		TMZ-free	3	0.76 (0.57–1.02)	0.068	0.748	40.8%	NA

Race	Caucasian	TMZ-containing	14	0.46 (0.34–0.63)	<0.001	0.014	76.2%	0.22
		TMZ-free	2	0.75 (0.41–1.38)	0.35	1	54.8%	NA
	Asian	TMZ-containing	5	0.49 (0.41–0.59)	<0.001	0.014	0%	NA
		TMZ-free	1	0.69 (0.50–0.94)	0.02	0.24	NA	NA
	Mixed race	TMZ-containing	3	0.51 (0.40–0.65)	<0.001	0.014	NA	NA
		TMZ-free	0	NA	NA	NA	NA	NA

Study type	non-RCT	TMZ-containing	21	0.47 (0.39–0.56)	<0.001	0.014	67%	0.19
		TMZ-free	3	0.76 (0.57–1.02)	0.07	0.7	40.8%	NA
	RCT	TMZ-containing	1	0.61 (0.52–0.73)	<0.001	0.014	NA	NA
		TMZ-free	0	NA	NA	NA	NA	NA

GBM type	Newly diagnosed	TMZ-containing	16	0.47 (0.39–0.57)	<0.001	0.014	66.1%	0.44
		TMZ-free	1	0.93 (0.70–1.24)	0.62	1	NA	NA
	Elderly	TMZ-containing	0	NA	NA	NA	NA	NA
		TMZ-free	0	NA	NA	NA	NA	NA
	Recurrent	TMZ-containing	6	0.49 (0.34–0.70)	<0.001	0.014	66%	NA
		TMZ-free	2	0.66 (0.49–0.88)	0.005	0.065	0%	NA

*HR, hazard ratio; CI, confidence interval; NA, not applicable; TMZ-containing treatment, TMZ-alone and combined radiotherapy/TMZ and combined radiotherapy/TMZ-containing chemotherapy; TMZ-free treatment, radiotherapy alone and combined radiotherapy/TMZ-free alkylation agents chemotherapy; Mixed race: patients in American studies; Bon, P for Step-down Bonferroni adjustment*.

### Association between MGMT Promoter Methylation and Survival in Newly Diagnosed GBM Subpopulation

**Figure 4:**
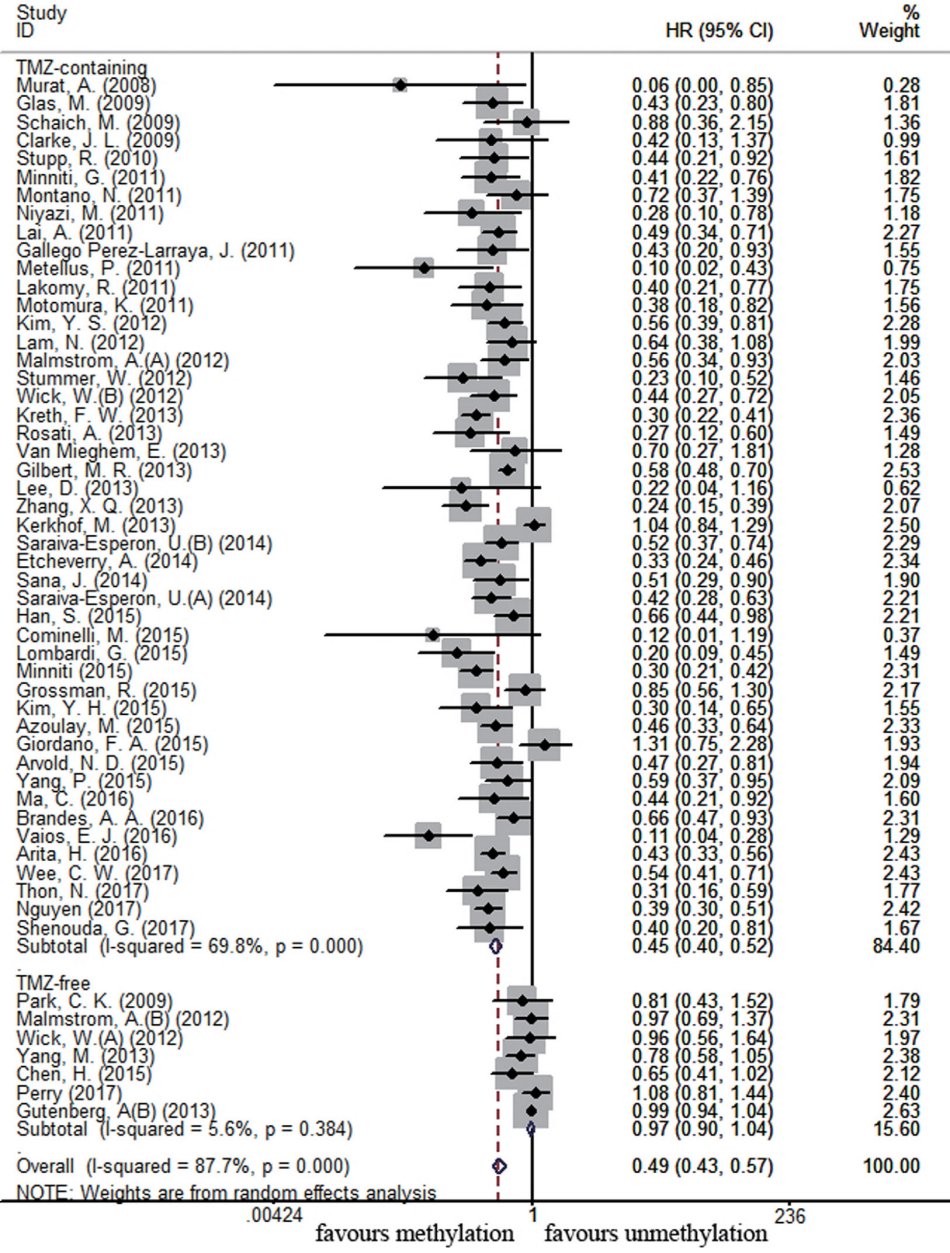
Calculated HRs and 95% CIs for the relationship between methylation and OS benefit from TMZ-containing or TMZ-free therapy in newly diagnosed GBM patients (methylated vs. unmethylated patients).

**Figure 5:**
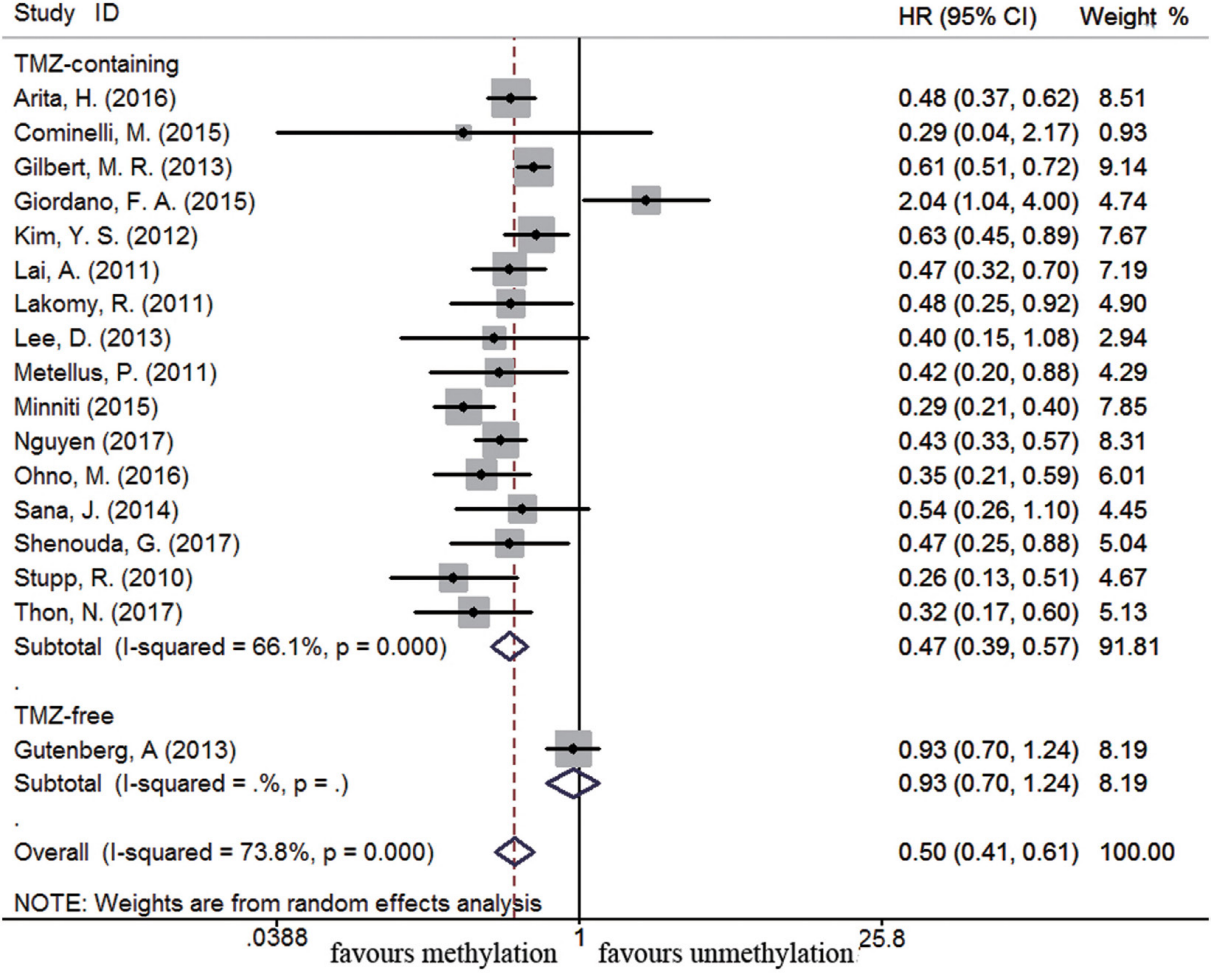
Calculated HRs and 95% CIs for the relationship between methylation and PFS benefit from TMZ-containing or TMZ-free therapy in newly diagnosed GBM patients (methylated vs. unmethylated patients).

There were 54 and 17 studies recruited to assess the impact of *MGMT* promoter methylation on OS and PFS in newly diagnosed GBM patients, respectively. *MGMT* promoter methylation in newly diagnosed GBM patients was also associated with improved OS and PFS (OS: HR = 0.49, 95% CI 0.43–0.57, *p* < 0.001, *I*^2^ = 87.7%; PFS: HR = 0.50, 95% CI 0.41–0.61, *p* < 0.001, *I*^2^ = 73.8%, Figure S2 in Supplementary Material). Subgroup analysis showed that methylated patients receiving TMZ-containing treatment had better OS and PFS than unmethylated patients (OS: HR = 0.45, 95% CI 0.40–0.52, p < 0.001, Bon = 0.017, *I*^2^ = 69.8%, Figure 4; PFS: HR = 0.47, 95% CI 0.39–0.57, *p* < 0.001, Bon = 0.014, *I*^2^ = 66.1%, Figure 5). No significant advantage on OS and PFS was observed in methylated patients receiving TMZ-free treatment (OS: HR = 0.97, 95% CI 0.90–1.04, *p* = 0.37, Bon = 1, *I*^2^ = 5.6%, Figure 4; PFS: HR = 0.93, 95% CI 0.70–1.24, *p* = 0.62, Bon = 1, Figure 5). These observations were similar to those in overall GBM patients, indicating that the beneficial effect of methylation on OS in newly diagnosed patients was also TMZ therapy-dependent.

### Association between *MGMT* Promoter Methylation and Survival in Elderly GBM Subpopulation

**Figure 6:**
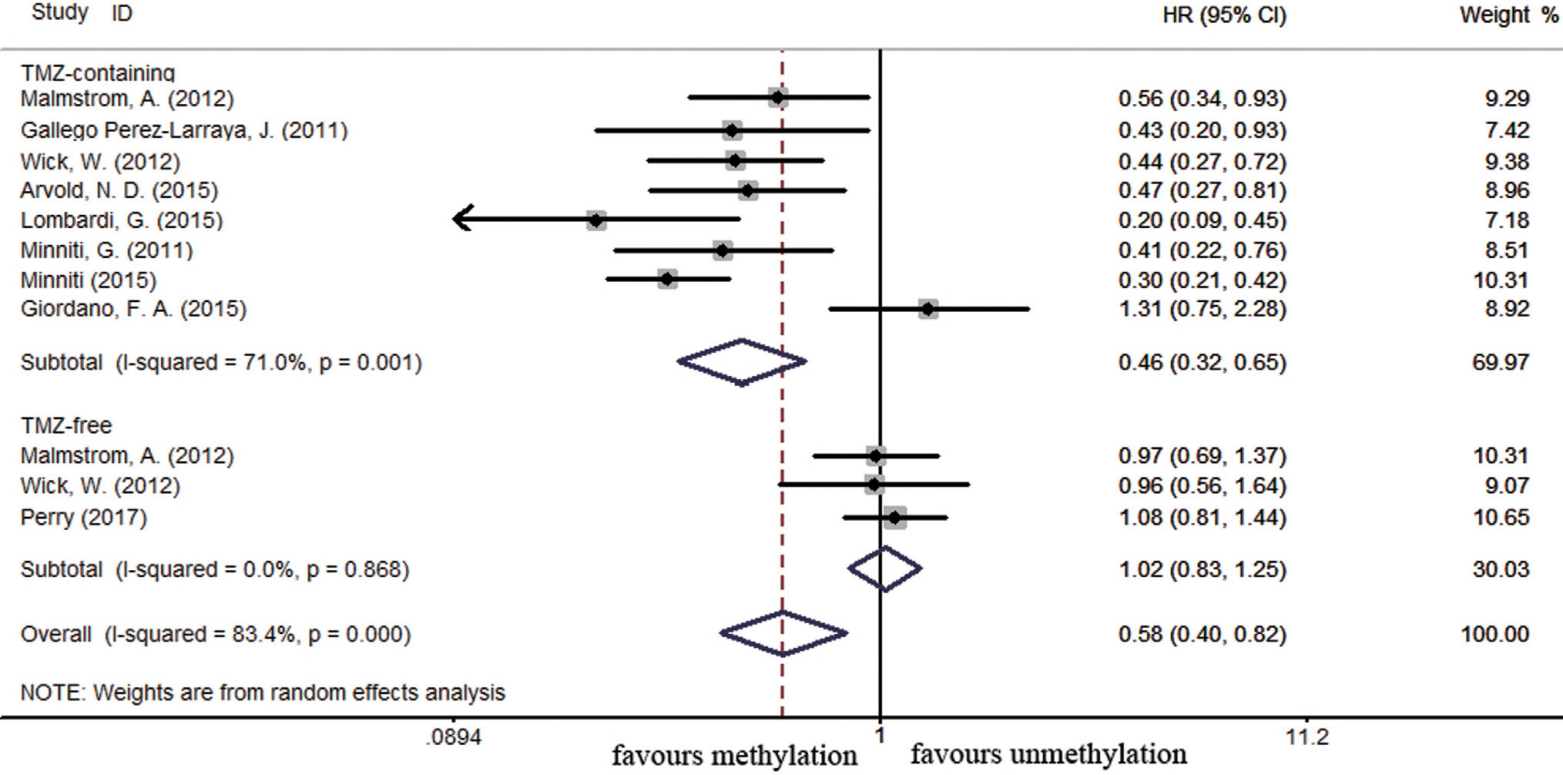
Calculated HRs and 95% CIs for the relationship between methylation and OS benefit from TMZ-containing or TMZ-free therapy in elderly GBM patients (methylated vs. unmethylated patients).

Overall survival in elderly GBM patients was assessed on the basis of 11 studies comprising 1,321 patients. Among these studies, elderly was defined as 60 years or older (58), over 65 years old (32, 41, 55, 61, 70, 84), or 70 years or older (39, 62). A significant correlation between *MGMT* promoter methylation and better OS was observed in elderly GBM patients (HR = 0.58, 95% CI 0.40–0.82, *p* = 0.002, *I^2^* = 83.4%, Figure S3 in Supplementary Material). A significant improvement on OS was also found in methylated elderly patients with TMZ-containing treatment compared to unmethylated patients with similar treatment (HR = 0.46, 95% CI 0.32–0.65, *p* < 0.001, Bon = 0.017, *I^2^* = 71%, Figure 6). No significance benefit from TMZ-free treatment found in methylated elderly patients than unmethylated elderly patients (HR = 1.02, 95% CI 0.83–1.25, *p* = 0.83, Bon = 1, *I*^2^ = 0%, Figure 6).

**Figure 7:**
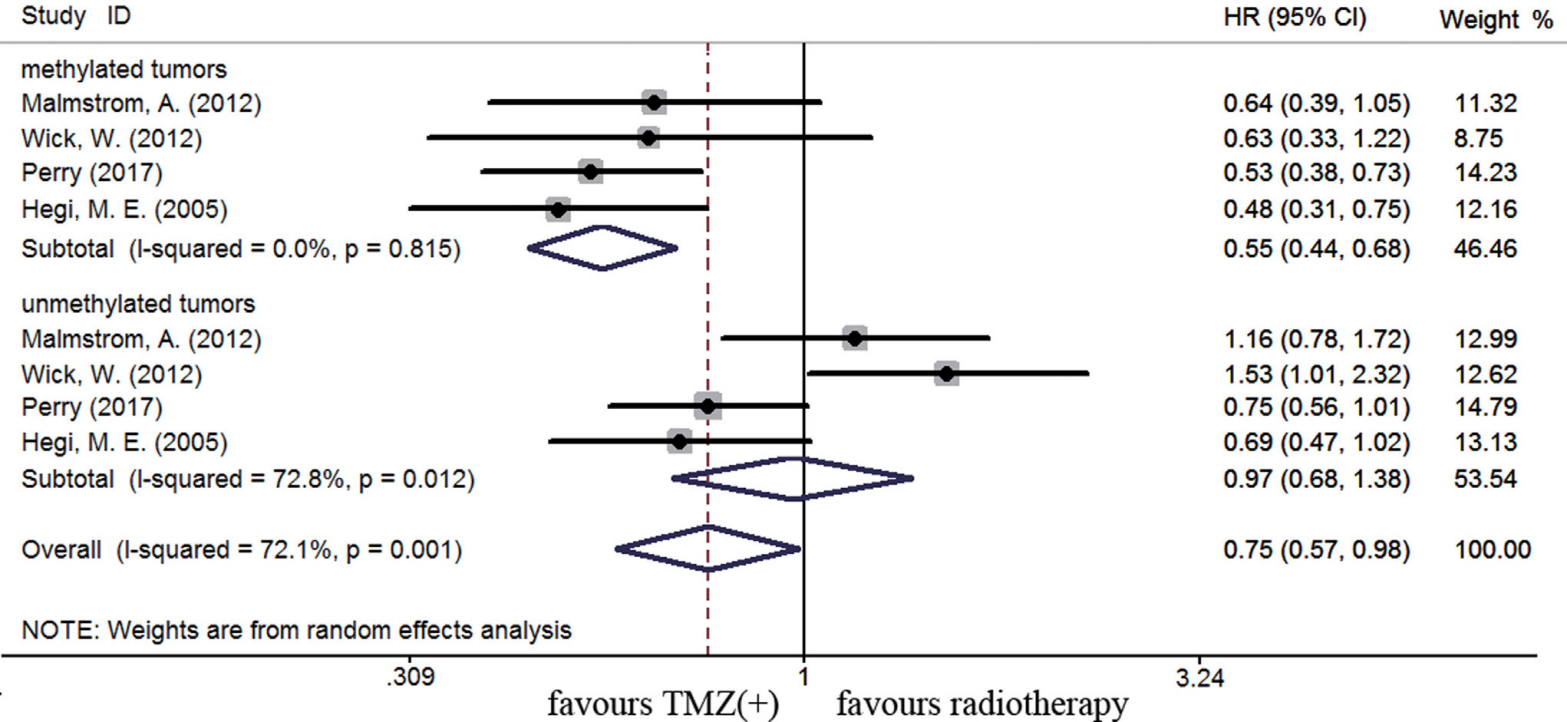
Calculated HRs and 95% CIs for the relationship between methylation and OS benefit in elderly GBM patients (TMZ-containing therapy vs. radiotherapy alone).

**Figure 8:**
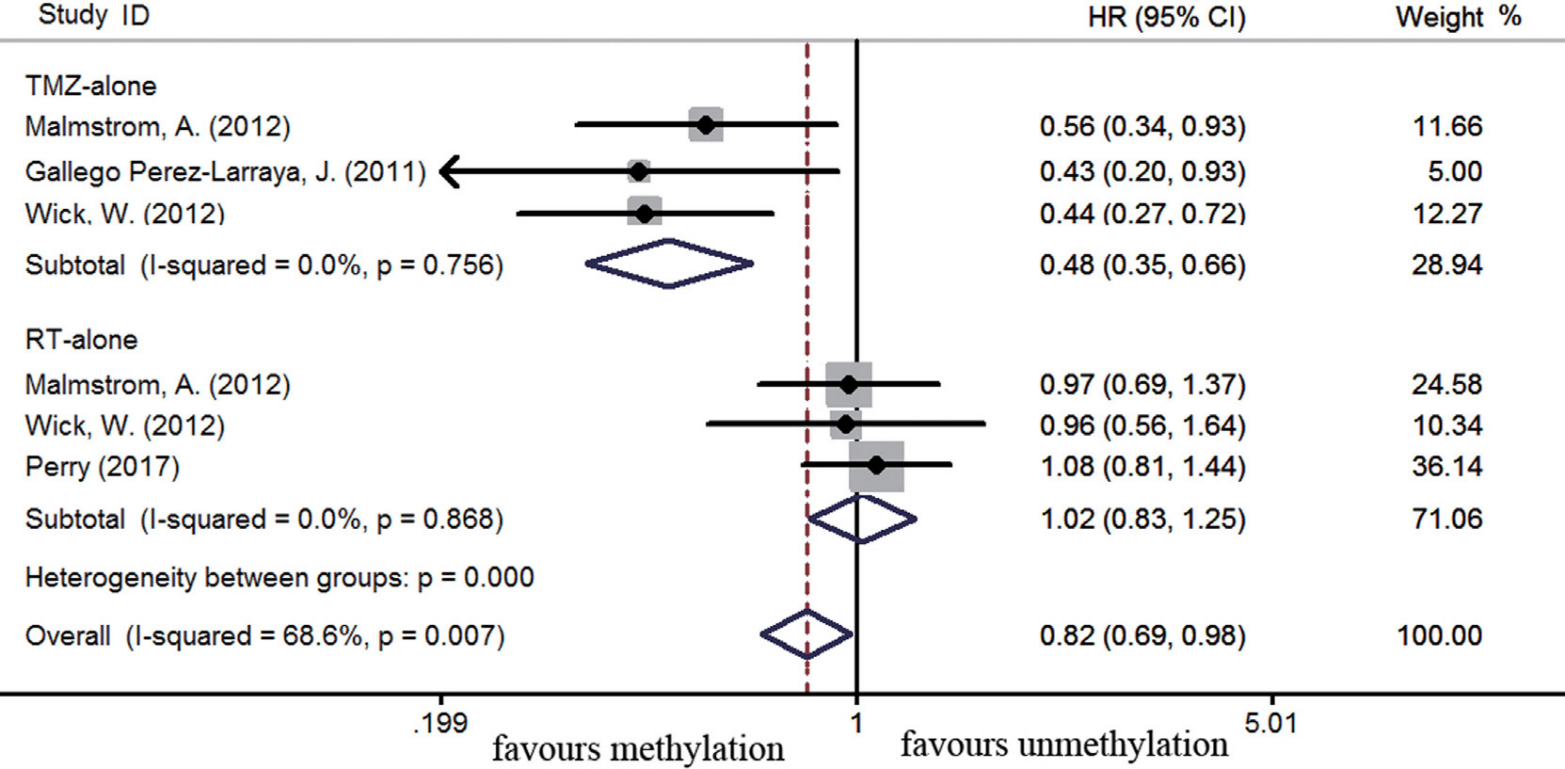
Calculated HRs and 95% CIs for the relationship between methylation and OS benefit in elderly GBM patients exposed to TMZ alone or radiotherapy (RT) alone (methylated vs. unmethylated patients).

The efficacy of TMZ-containing therapy versus radiotherapy in elderly patients was assessed according to three randomized controlled trials (58, 70, 84). Methylated elderly patients with TMZ-containing treatment had better OS than those with radiotherapy alone (HR = 0.55, 95% CI 0.44–0.68, *p* < 0.001; *I^2^* = 0%, Figure 7). However, the benefit of TMZ-containing therapy was not observed in elderly patients with unmethylated status (HR = 0.97, 95% CI 0.68–1.38, *p* < 0.001, *I*^2^ = 72.8%, Figure 7). Elderly patients were often unable to tolerate multimodality therapy, so we further assess whether elderly patients with *MGMT* methylation could benefit from TMZ alone or radiotherapy alone therapy. Compared to unmethylated elderly patients, prolonged OS was observed in methylated elderly patients receiving TMZ alone therapy but not in those receiving radiotherapy alone (TMZ alone: HR = 0.48, 95% CI 0.35–0.66, *p* < 0.001, *I*^2^ = 0%; Radiotherapy alone: HR = 1.02, 95% CI 0.83–1.25, *p* = 0.83, *I*^2^ = 0%, Figure 8). These results indicated the strong correlation between *MGMT* methylation and better response to TMZ therapy in elderly GBM patients.

### Association between *MGMT* Promoter Methylation and Survival in Recurrent GBM Subpopulation

**Figure 9:**
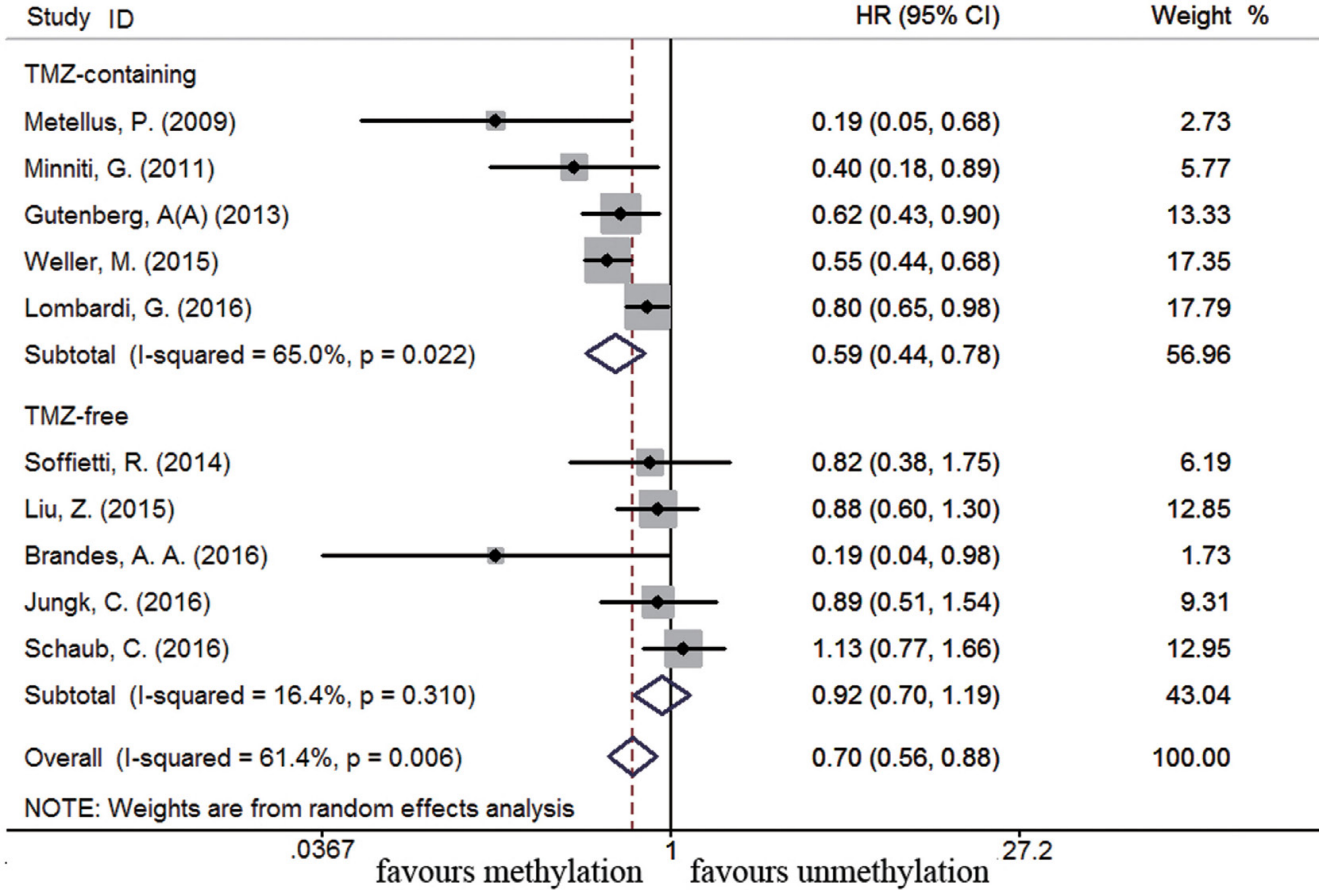
Calculated HR and 95% CIs for the relationship between methylation and OS benefit from TMZ-containing or TMZ-free therapy in recurrent GBM patients (methylated vs. unmethylated patients).

**Figure 10:**
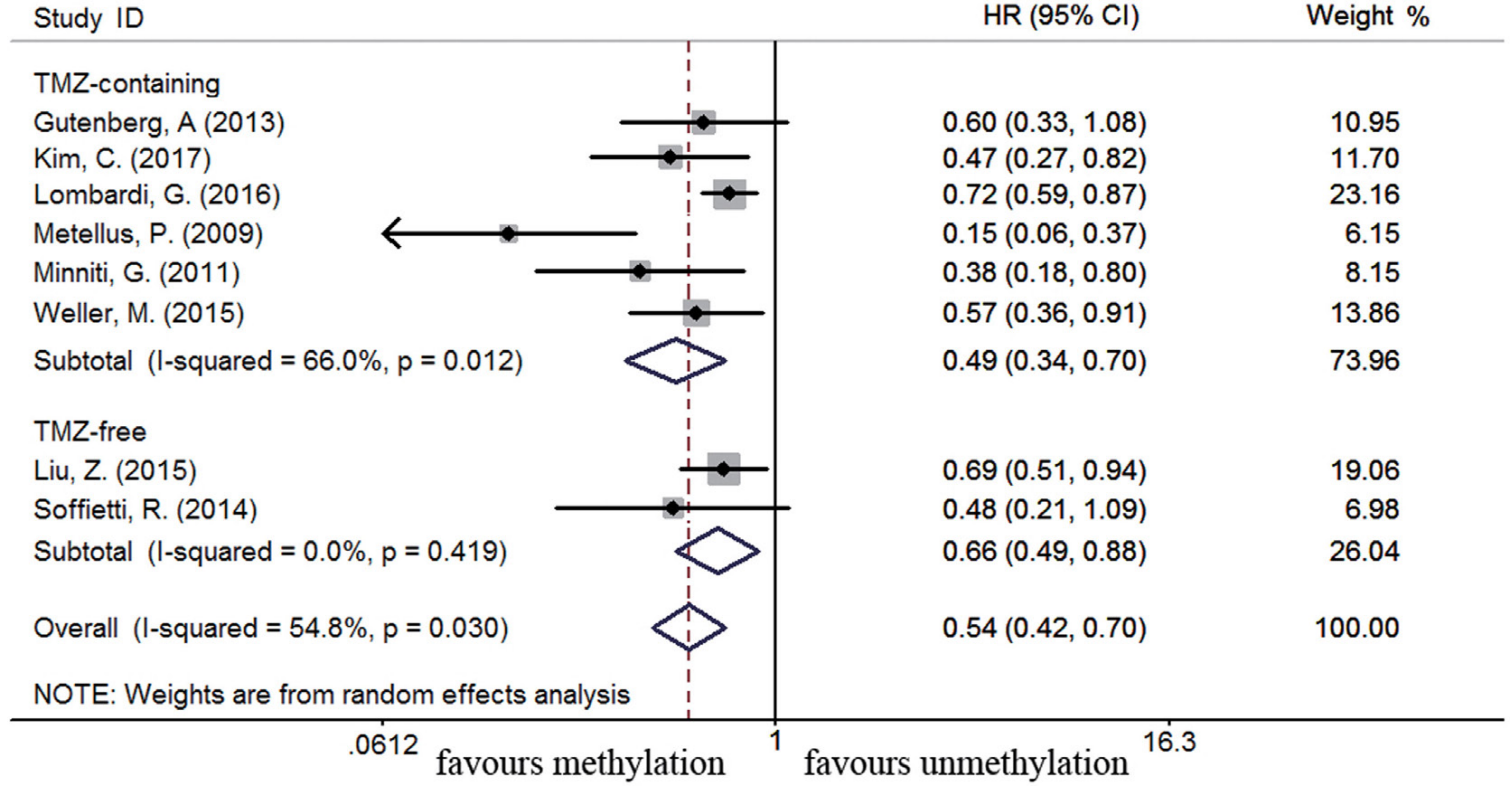
Calculated HR and 95% CIs for the relationship between methylation and PFS benefit from TMZ-containing or TMZ-free therapy in recurrent GBM patients (methylated vs. unmethylated patients).

Eleven studies were included to analyze the association between *MGMT* promoter methylation and survival in recurrent GBM patients (19, 21, 22, 44, 46, 56, 60, 63, 75, 77, 89). A significant improvement on OS and PFS was observed in methylated recurrent patients (OS: HR = 0.70, 95% CI 0.56–0.88, *p* < 0.001, *I*^2^ = 61.4%; PFS: HR = 0.54, 95% CI 0.42–0.70, *p* < 0.001, *I*^2^ = 54.8%, Figure S4 in Supplementary Material). Subgroup analysis showed TMZ-containing therapy conferred a survival benefit in methylated recurrent patients (OS: HR = 0.59, 95% CI 0.44–0.78, *p* < 0.001, Bon = 0.017, *I*^2^ = 65%, Figure 9; PFS: HR = 0.49, 95% CI 0.34–0.70, *p* = 0.001, Bon = 0.014, *I*^2^ = 66%, Figure 10). In contrast, TMZ-free therapy did not improve OS (HR = 0.92, 95% CI 0.70–1.19, *p* = 0.52, Bon = 1, *I*^2^ = 16.4%, Figure 9) or PFS (HR = 0.66, 95% CI 0.49–0.88, *p* = 0.005, Bon = 0.065, *I*^2^ = 0%, Figure 10) in methylated recurrent patients.

### Association between *MGMT* Promoter Methylation and Survival in GBM Patients with Different Races

There were 42 studies for Caucasian (European, Canadian, Australian), 16 studies for Asian (Chinese, Japanese, Korean), and 8 studies for mixed race (American). Compared to unmethylated patients, both OS and PFS were improved in methylated patients (OS: Asian: HR = 0.54, 95% CI 0.44–0.65, p < 0.001, *I^2^* = 61.1%; Caucasian: HR = 0.53, 95% CI 0.45–0.63, p < 0.001, *I^2^* = 86.8%; Mixed race: HR = 0.48, 95% CI 0.38–0.62, p < 0.001, *I*^2^ = 67.7%; PFS: Asian: HR = 0.53, 95% CI 0.43–0.65, p < 0.001, *I^2^* = 31.4%; Caucasian: HR = 0.49, 95% CI 0.37–0.65, *p* < 0.001, *I^2^* = 77.8%; Mixed race: HR = 0.51, 95% CI 0.40–0.65, p < 0.001, *I^2^* = 61%, Figure S5 in Supplementary Material). Among GBM patients with TMZ-containing treatment, *MGMT* methylation benefited to both Caucasian and Asian (Asian OS: HR = 0.48, 95% CI 0.42–0.54, *p* < 0.001, Bon = 0.017, *I*^2^ = 43.8%; PFS: HR = 0.49, 95% CI 0.41–0.59, *p* < 0.001, Bon = 0.014, *I*^2^ = 0%; Caucasian OS: HR = 0.46, 95% CI 0.39–0.55, *p* < 0.001, Bon = 0.017, *I*^2^ = 75.5%; PFS: HR = 0.46, 95% CI 0.34–0.63, *p* < 0.001, Bon = 0.014, *I*^2^ = 76.2%, Figure S6 in Supplementary Material). Among patients receiving TMZ-free treatment, survival benefit in Asian patients was not observed anymore after Bonferroni correction (Asian OS: HR = 0.78, 95% CI 0.64–0.95, *p* = 0.02, Bon = 0.24, *I*^2^ = 0%; PFS: HR = 0.69, 95% CI 0.50–0.94, *p* = 0.02, Bon = 0.24, Figure S6 in Supplementary Material). No benefit was observed in Caucasian receiving TMZ-free therapy regardless of Bonferroni adjustment. The impact of *MGMT* promoter methylation in mixed race was not evaluated since data in TMZ-free group was not available.

### Publication Bias

Publication bias was evaluated by Egger’s test. Publication bias was observed in OS and PFS analysis in overall GBM patients (OS: *p* < 0.001; PFS: *p* = 0.04). More results were presented in Table 2. Therefore, we performed the trim and fill analysis to estimate the publication bias. However, those results remain unchanged after introducing the trim and fill method to correct the publication bias.

### Sensitive Analysis

Sensitivity analysis was conducted by sequentially omitting individual studies to assess whether a single study might significantly affect the overall results. Sensitivity analysis showed one study (41) predominantly contributed to heterogeneity in elderly GBM subpopulation, especially in TMZ-containing group (Figure S7 in Supplementary Material). Further sensitivity analysis revealed that other results did not show any apparent variations in pooled HRs for OS or PFS, supporting the robustness of the primary findings.

## Discussion

Although MGMT has been widely established as a clinically relevant biomarker in GBM patients, its clinical implication has not been definitely confirmed. A prognostic factor is a clinical or biologic characteristic that is objectively measured and provides information on likely outcome of the cancer disease independent of treatment, while a predictive factor is a clinical or biologic characteristic providing information on likely benefits from one specific treatment rather than another (90). Which one is more appropriate to describe the relationship between *MGMT* promoter methylation and GBM prognosis? Among overall GBM patients, *MGMT* methylation conferred a survival benefit to patients with TMZ-containing treatment, but not to those with TMZ-free treatment. It seems that *MGMT* methylation has a predictive value for GBM patients exposed to TMZ-containing treatment. However, considering the differentiation of prognostic variables among patients, including primary or recurrent GBM, age and race, the universality of predictive value of *MGMT* methylation in different GBM subgroups should be profoundly validated. Therefore, we further assess its clinical significance in newly diagnosed patients, recurrent patients, elderly patients, and Asian and Caucasian patients.

In newly diagnosed and recurrent GBM patients, *MGMT* methylation was associated with improved OS and PFS with TMZ-containing treatment, but not in those with TMZ-free treatment. Then *MGMT* methylation is predictive for a benefit from TMZ–containing chemotherapy in newly diagnosed and recurrent patients.

In elderly GBM patients, *MGMT* methylation also conferred an OS benefit in patients with TMZ-containing treatment, but not in those with TMZ-free treatment. Therefore, *MGMT* methylation in elderly patients is likely to have a similar predictive value as in newly diagnosed and recurrent GBM patients. Elderly GBM patients are often clinically unable to tolerate multimodality therapy, thus TMZ or radiotherapy alone is commonly used. This meta-analysis showed that elderly patients with methylated status exposed to TMZ alone had improved OS than those exposed to radiotherapy alone, while such difference was not observed in those with unmethylated status. Our results highlight that TMZ alone therapy might be a more effective option than radiotherapy alone therapy for elderly GBM patients with methylated *MGMT* status. But the optimal radiotherapy regimen for elderly and/or frail patients with newly diagnosed GBM remains to be defined (91). A recent study showed that short-course radiation (40 Gy in 15 fractions) plus TMZ conferred a survival advantage over radiotherapy alone in elderly patients (65 years of age or older) with newly diagnosed GBM, especially in those with methylated *MGMT* status (70). Due to the lack of a uniform definition for elderly, different cutoff age was employed in different studies. Patients aged more than 70 years were excluded from Stupp study (87). In this meta-analysis, patients aged 60 or more were enrolled for analysis. Our results showed that patients aged over 70 years with *MGMT* methylation also benefit from TMZ-containing therapy. The definition of cutoff age for the elderly are closely linked to prognosis, therapeutic goals, or patterns of care, so further research in this field should standardize the cutoff age for enrollment eligibility (92).

Another interesting issue is the clinical value of *MGMT* methylation in Asian and Caucasian patients. A previous study showed that *MGMT* methylation correlated with better OS and PFS in Caucasian patients and only better OS in Asian patients regardless of therapeutic intervention (93). But the benefit of different therapies in methylated patients was not investigated in the study. In our analysis, survival benefit in Asian patients with TMZ-free treatment was not observed anymore after Bonferroni adjustment. Bonferroni correction can avoid false positives, and then the risk of false negatives would be increased. So the finding in Asian patients should be cautiously interpreted. It must be noted that only four studies (519 patients) for OS and a single study (137 patients) for PFS were enrolled for this subgroup analysis. Therefore, our finding on patients with different races needs to be further verified by more clinical studies. Furthermore, recent studies also give a hint about the different regulation of *MGMT* methylation in different ethnic background. Single nucleotide polymorphisms (rs16906252) in *MGMT* promoter-enhancer is a key determinant in the acquisition of *MGMT* methylation (94). The genotype of rs16906252 varies among different ethnic groups (95), which may result in different *MGMT* methylation status. In addition to promoter methylation, other molecules are also involved in regulation of MGMT expression or function. For example, miR-181d can bind to the 3′untranslated region of *MGMT* transcripts, then decrease its mRNA stability and/or reduce protein translation (96). Further studies on ethnically genetic variations are necessary.

Due to the limited number of trials recruited for analysis, the presented information about PFS in patients with TMZ-free treatment, especially in newly diagnosed and recurrent subgroups, should be interpreted carefully. It should be acknowledged that we did not obtain any data of PFS in elderly patients exposed to TMZ-free treatment. Therefore, the predictive or prognostic value of this biomarker for PFS is far from identified in our analysis. In fact, clinical measurement of PFS may be a critical challenge in GBM trials. It is well known that GBM patients suffer inevitably recurrence despite integrated therapy (97). Pseudoprogression, also denoted as radiotherapy-introduced necrosis, exhibits contrast enhancement similar to early tumor progression on magnetic resonance imaging. Primary GBM patients receiving concurrent and adjuvant TMZ-based chemoradiotherapy have a high likelihood of developing pseudoprogression (98, 99), which occurs mainly within 3 months after completion of chemoradiotherapy. However, no technique has been proven to reliably differentiate between tumor recurrence and pseudoprogression. Additionally, both entities might coexist in the same patient at the same time in different areas of the tumor. The misdiagnosis of pseudoprogression as tumor recurrence may lead to a record of shorter PFS. Interestingly, *MGMT* promoter methylation was associated with a high incidence of pseudoprogression in newly diagnosed GBM patients undergoing TMZ-based chemoradiotherapy (100). In addition, GBM patients with the occurrence of pseudoprogression had a longer OS than those without pseudoprogression (98, 101), indicating that pseudoprogression may be a predictor for better response to therapy. Therefore, it is critically important to develop imaging techniques and biomarkers to discriminate pseudoprogression from early progression.

We also noticed the methodological diversity of measurement of *MGMT* promoter methylation. *MGMT* promoter methylation was detected by methylation-specific polymerase chain reaction (MSP), pyrosequencing, and methylation-sensitive high-resolution melting (MS-HRM) in 48, 6, and 2 studies, respectively. Additionally, various cutoff values for methylated positivity were used in these studies. However, there were few studies that have compared the merits and disadvantages of these *MGMT* testing methods (17). Further efforts should standardize the MGMT methylation testing methods and cutoff point.

Limitations of this study should be acknowledged. Firstly, heterogeneity existed in the pooled analysis for PFS and OS either in overall population or in subgroup analysis. Heterogeneity may result from different techniques of defining *MGMT* promoter status and varied therapy schedule. Different chemotherapy and radiotherapy schedules may influence the prognosis of GBM patients, thus analysis of the correlation between a single treatment schedule and *MGMT* promoter status was not conducted in this meta-analysis. Second, considering the scarce number of multivariate studies in some of subgroup analysis, univariate studies were also included in our analysis. We also performed analysis using only multivariate studies and similar findings were observed (Table S3 in Supplementary Material). Third, due to the limited number of original documents on PFS, there was not enough power to identify the impact of *MGMT* methylation on PFS, especially in patients receiving TMZ-free therapy. Fourth, quality assessment was performed by a modified domain-based NOS (102, 103), which was proposed as a potential helpful and practically method for assessment of tumor prognostic studies. However, this novel NOS has not been fully validated and results should be interpreted with caution. Fifth, Egger’s test showed that publication bias existed in pooled analysis for OS, but the trim and fill analysis upheld the reliability of our results.

In conclusion, our results highlight the universal predictive value of *MGMT* methylation in newly diagnosed GBM patients, elderly GBM patients and recurrent GBM patients. For elderly methylated GBM patients, TMZ alone therapy might be a more suitable option than radiotherapy alone therapy. This study may be helpful to optimize therapeutics in different GBM subpopulation.

## Author Contributions

Y-HZ and C-JC contributed to the conception of the experiments and manuscript preparation. Y-HZ, C-SX, X-TZ, J-LL, JL, and KL contributed to data research and review. Y-HZ and HW performed data analysis. Z-FW and Z-QL contributed to interpretation and discussion of the results.

## Conflict of Interest Statement

The authors declare that the research was conducted in the absence of any commercial or financial relationships that could be construed as a potential conflict of interest. The reviewers CS, BW and handling Editor declared their shared affiliation.
