# Supplementary material for: The Clinical Significance of O6-Methylguanine-DNA Methyltransferase Promoter Methylation Status in Adult Patients With Glioblastoma: A Meta-analysis
Source: Front Neurol. 2018 Mar 21;9:127. doi: 10.3389/fneur.2018.00127 (PMC5873285; doi:10.3389/fneur.2018.00127)
Supplement: Supplementary file 1 [file data_sheet_1.docx]

**The Clinical Significance of MGMT Promoter Methylation Status in Adult Patients with Glioblastoma: A Meta-analysis**

Yu-Hang Zhao^1#^, Ze-Fen Wang^2#^, Chang-Jun Cao^1^, Hong Weng^3^, Cheng-Shi Xu^1^, Kai Li^1^,Jie-Li Li^1^, Jing-Lan^1^, Xian-Tao Zeng^3^, Zhi-QiangLi^1*^

1Department of Neurosurgery, Zhongnan Hospital, Wuhan University, Wuhan, China

2Department of Physiology, School of Basic Medical Sciences, Wuhan University, Wuhan, China

3Center for Evidence-based and Translational Medicine, Zhongnan Hospital, Wuhan

University, Wuhan, China

# Equal contributors

*Corresponding author:

**Zhi-Qiang Li**,

Department of Neurosurgery, Zhongnan Hospital of Wuhan University E-mail: [lizhiqiang@whu.edu.cn](mailto:lizhiqiang@whu.edu.cn)

**Supplementary Figures**

**Fig. S1** Calculated HRs and 95% CIs for the impact of methylation on OS (a) and PFS (b) in overall GBM patients (methylated *vs* un-methylated).

**a**


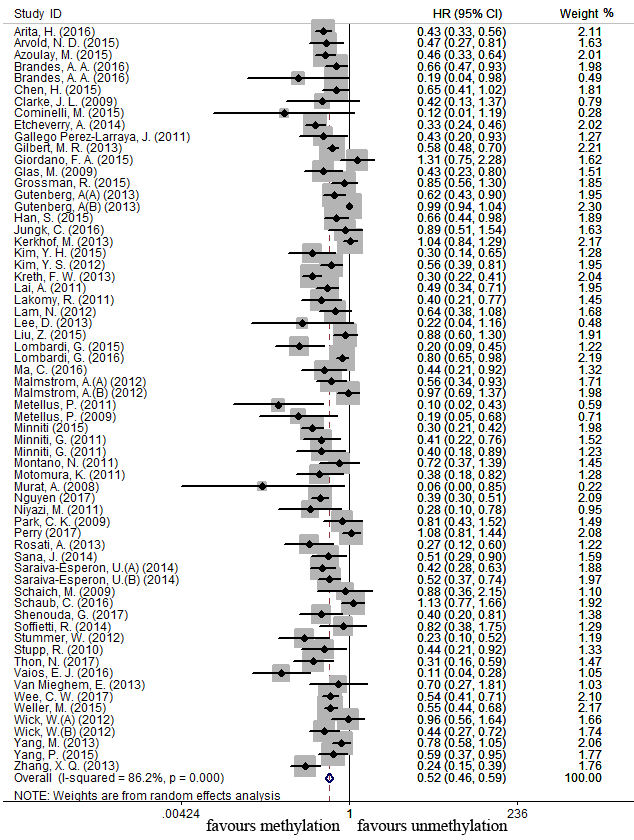


**b**

**
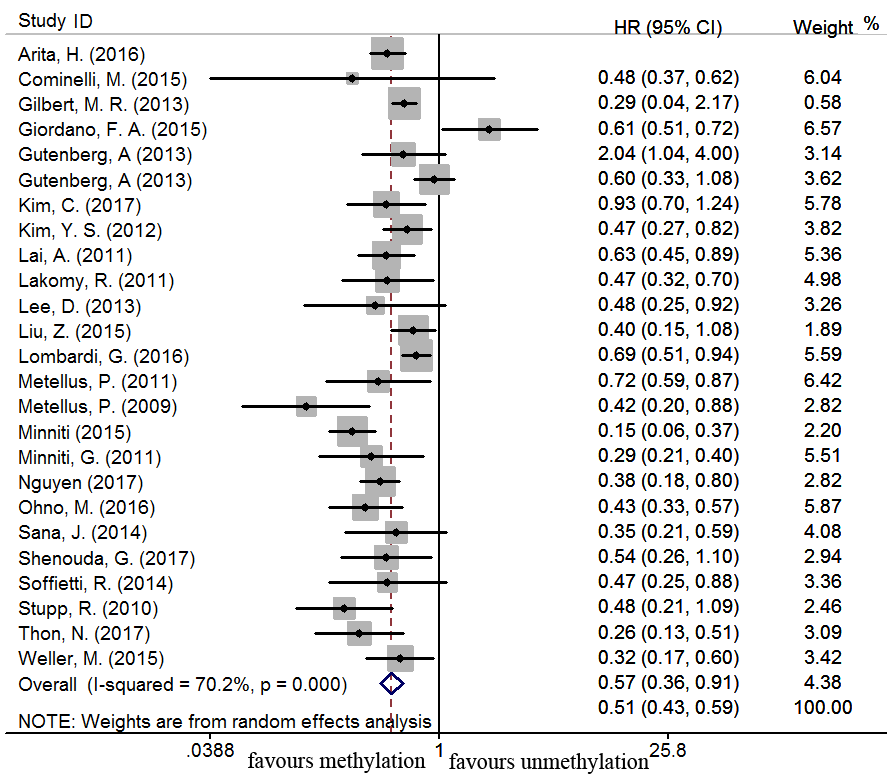
**

**Fig. S2** Calculated HRs and 95% CIs for the impact of methylation on OS (a) and PFS (b) in newly diagnosed GBM patients (methylated *vs* un-methylated).

a


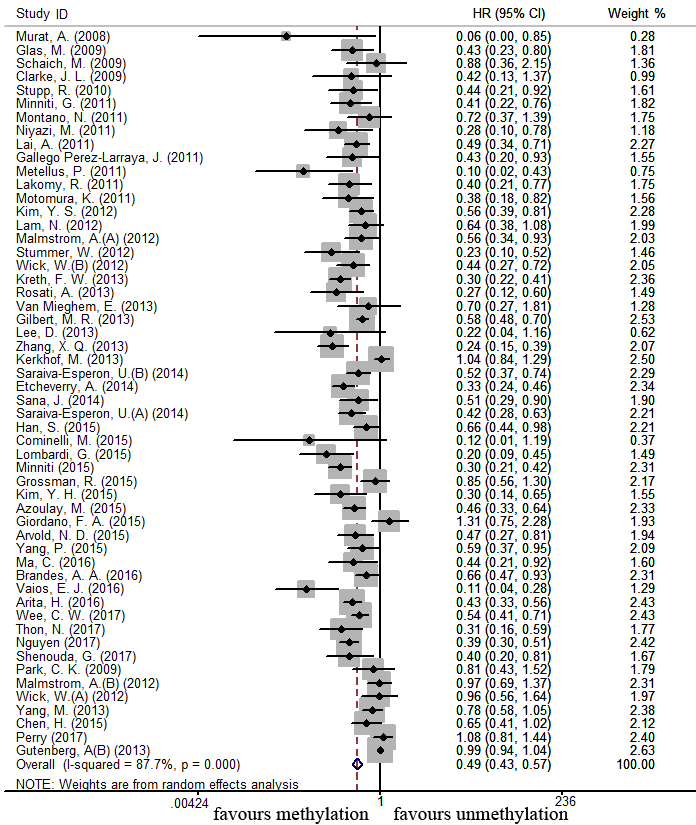


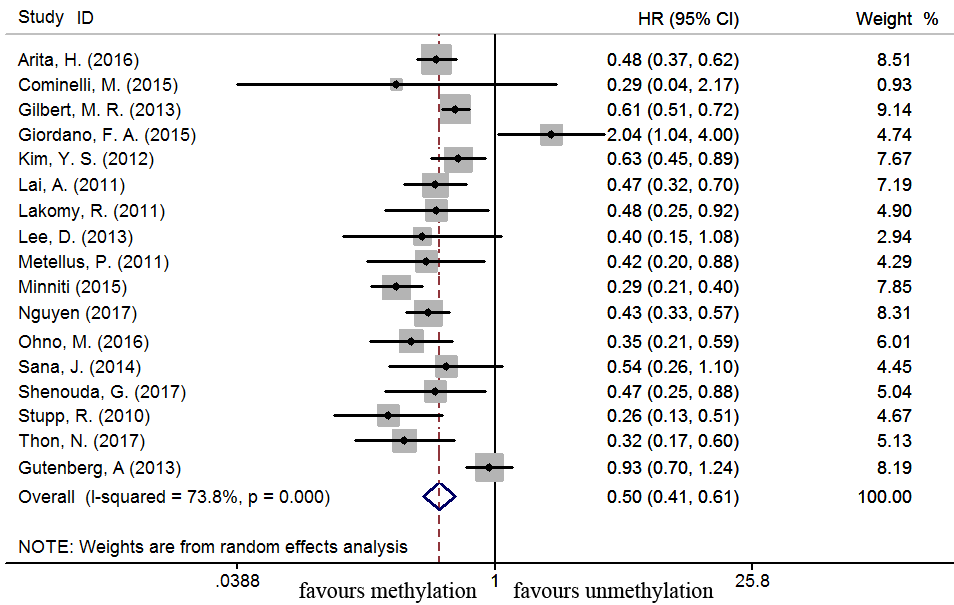


**Fig. S3** Calculated HRs and 95% CIs for the impact of methylation on OS in elderly GBM patients (methylated *vs* un-methylated).


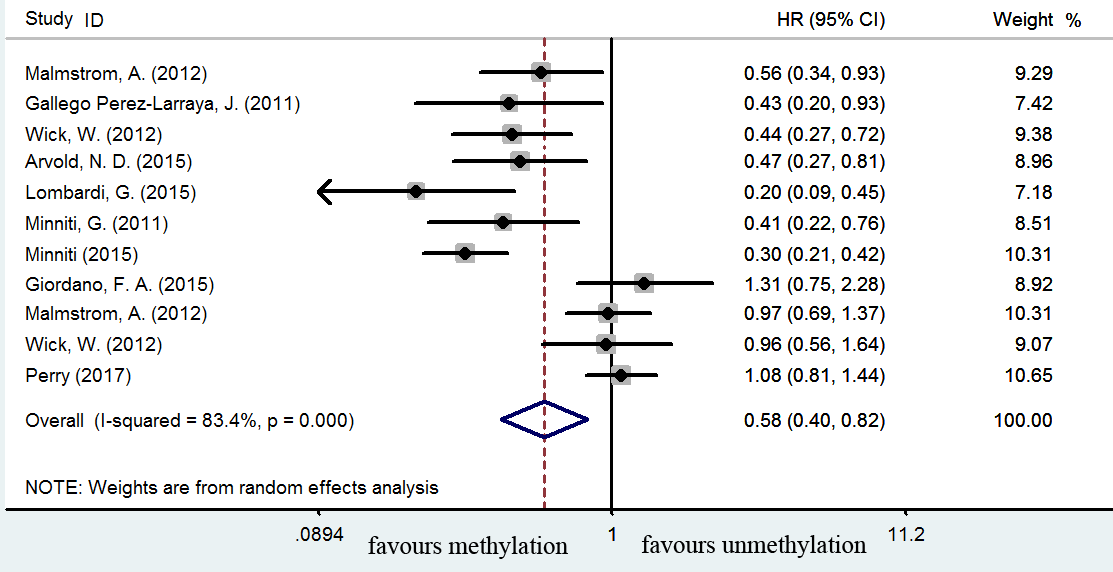
.

**Fig. S4** Calculated HRs and 95% CIs for the impact of methylation on OS (a) and PFS (b) in recurrent GBM patients (methylated *vs* un-methylated)

**a**

**
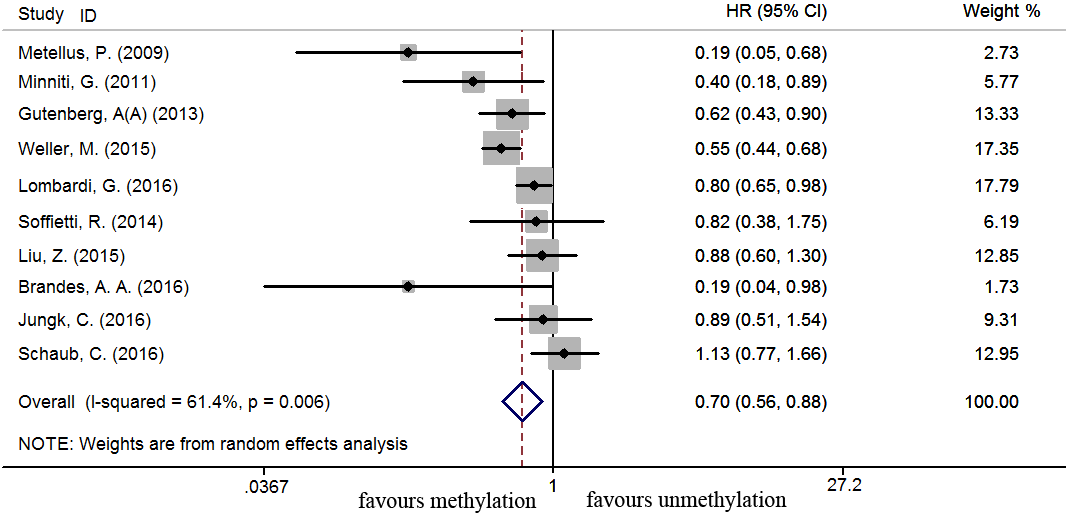
**

**b**

**
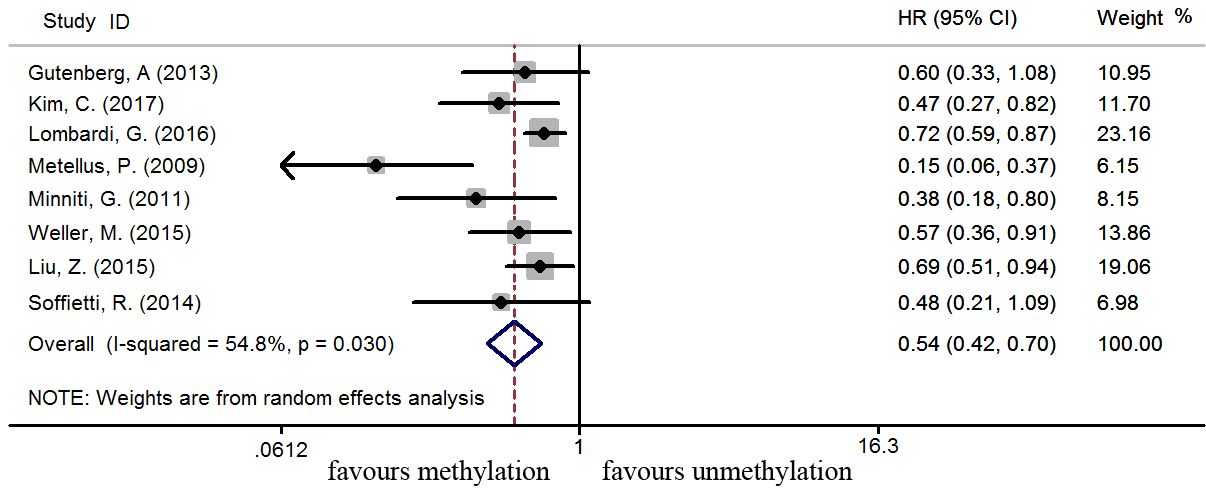
**

**Fig. S5** Calculated HRs and 95% CIs for the impact of methylation on OS (a) and PFS (b) in Asian ，Caucasian and mixed race GBM patients (methylated *vs* un-methylated patients)

**a**


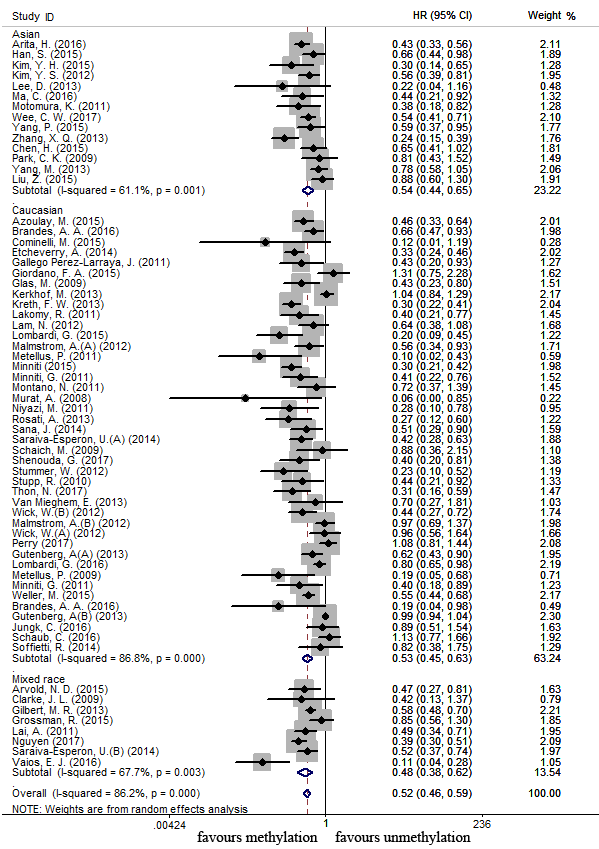


**B**

**
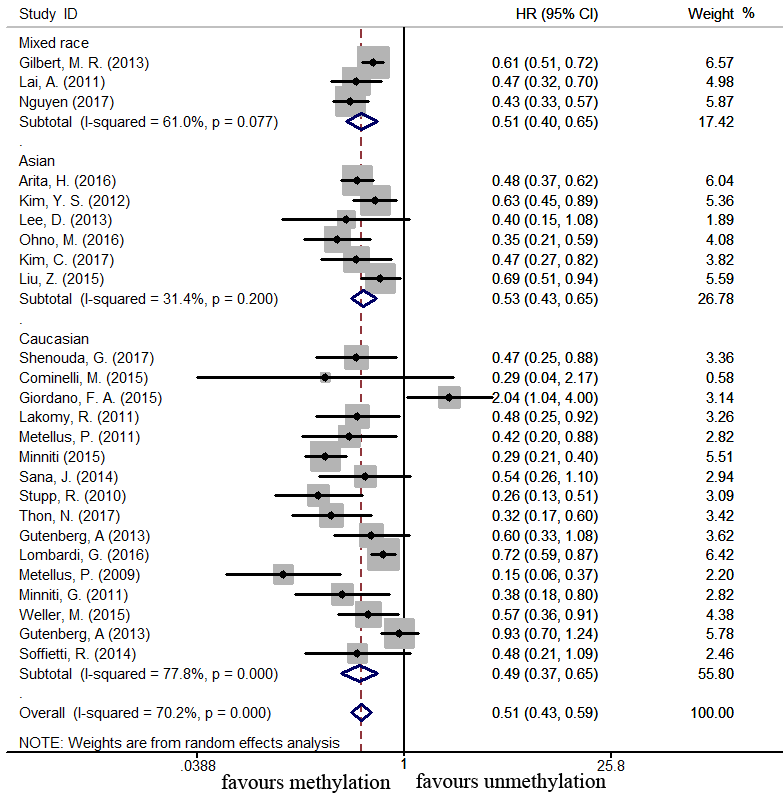
**

**Fig. S6** Calculated HRs and 95CIs for the impact of methylation on OS (a, c, e) and PFS (b, d) benefit from TMZ-containing or TMZ-free therapy in Asian (a, b), Caucasian(c, d) and mixed race (e) GBM patients (methylated *vs* un-methylated patients).

a


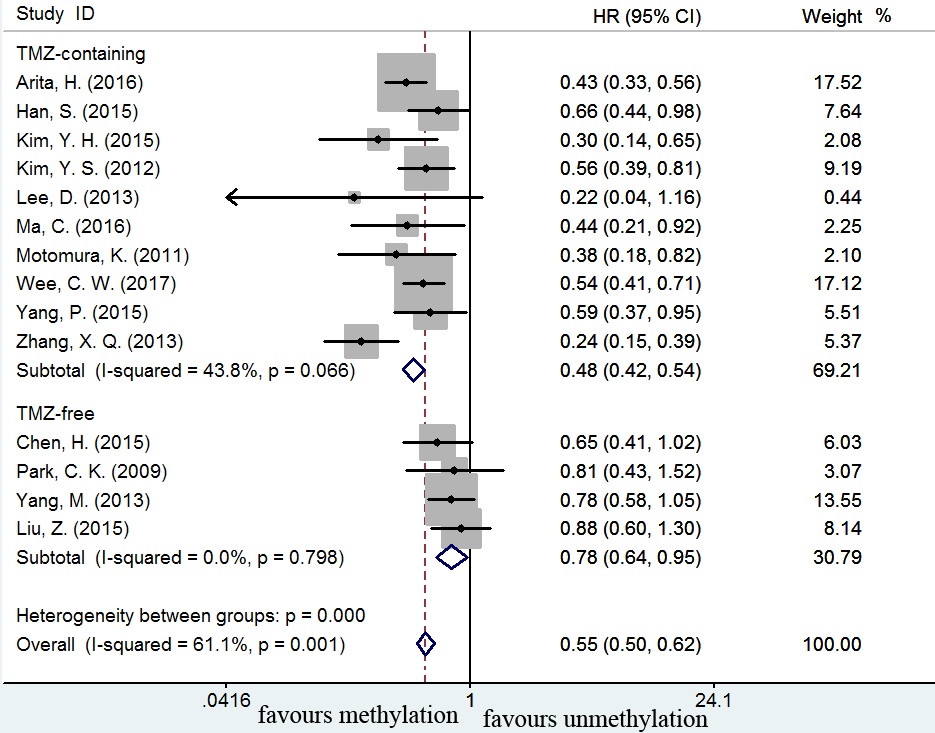


b


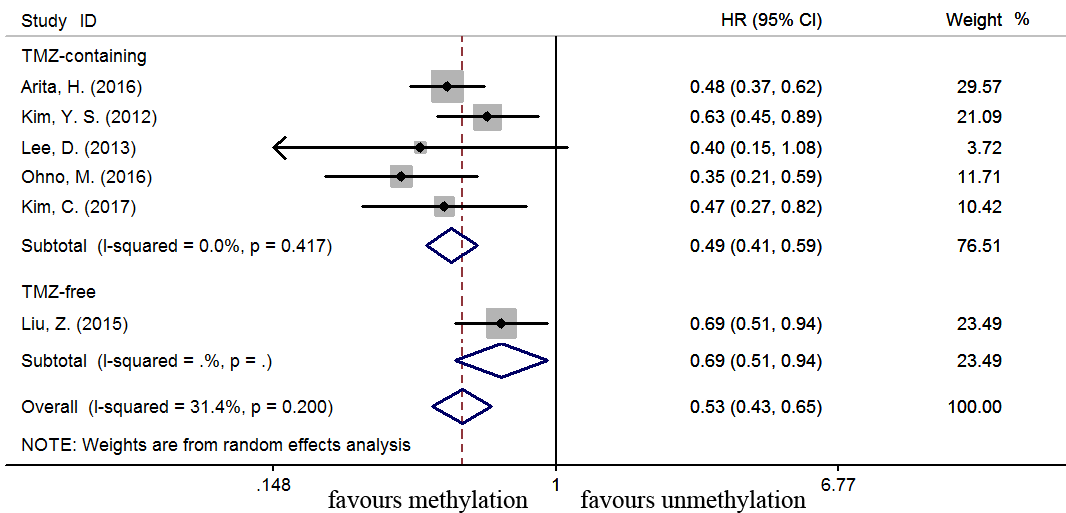


**c**


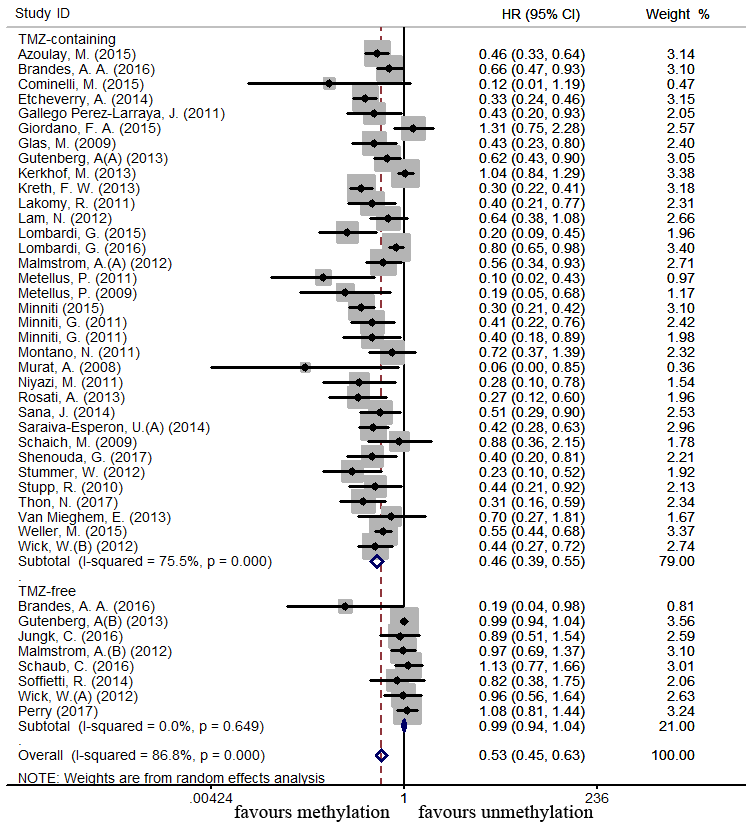


d


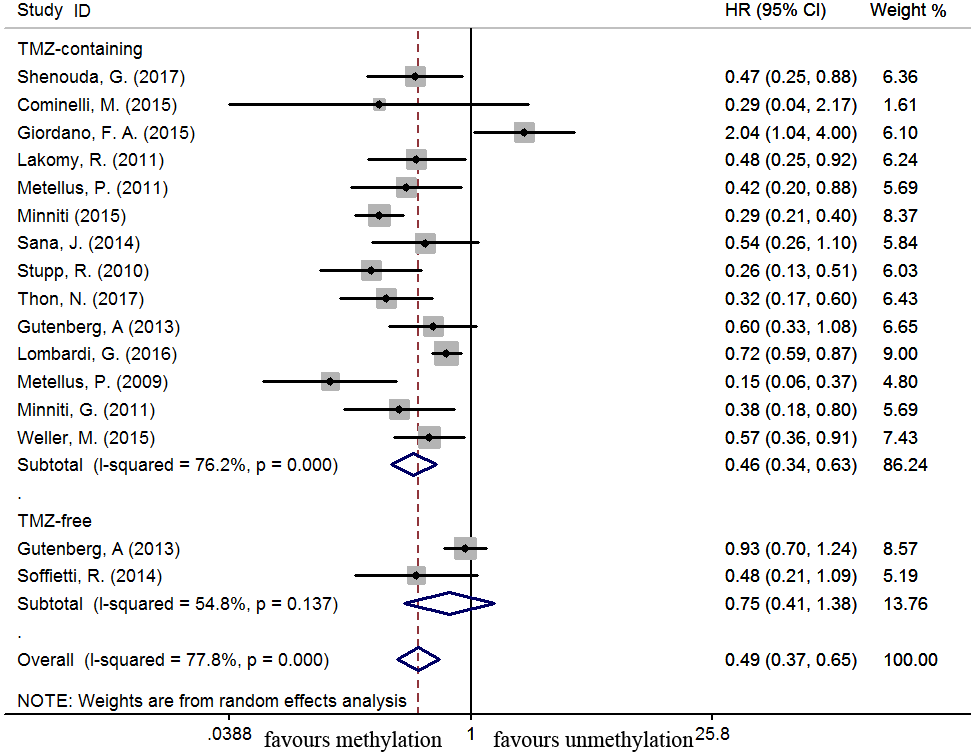


e


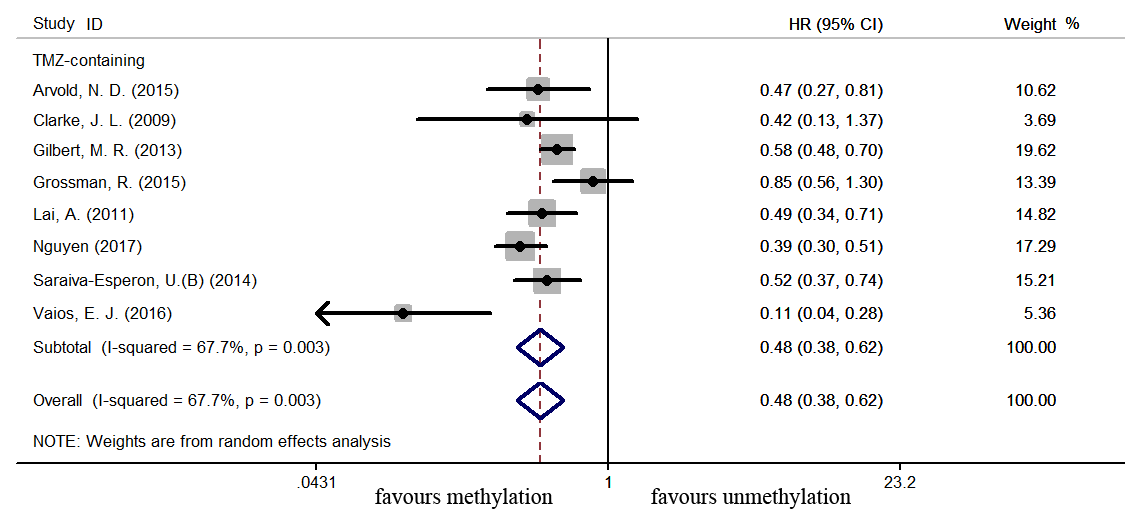


**Fig. S7: Sensitivity analysis**


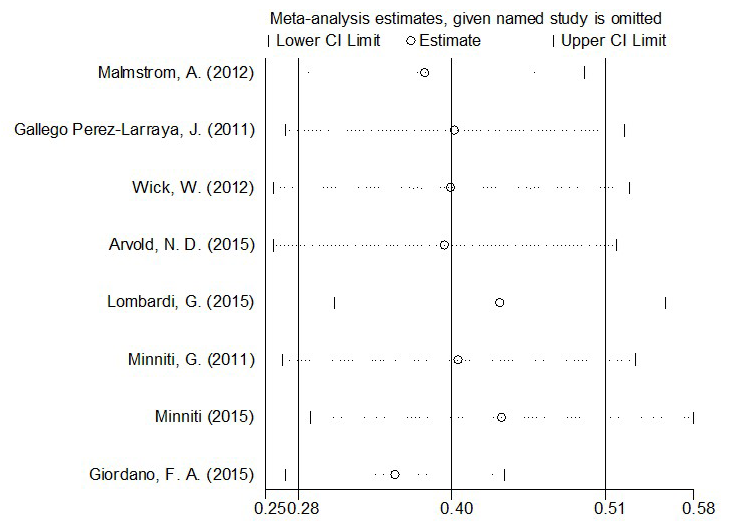


# Supplementary Table S1. Assessment of bias risk

| ***Domains*** | Minniti, G. | Arvold, N.D. | Azoulay, M. | Wee, C. W. | Chen, H. | Cominelli, M. | Etcheverry, A. |
| --- | --- | --- | --- | --- | --- | --- | --- |
|  | 2011(GBM) | 2015 | 2015 | 2017 | 2015 | 2015 | 2014 |
| ***Selection bias*** |  |  |  |  |  |  |  |
| *1. Selection* |  |  |  |  |  |  |  |
| 1.1 Treatment assignment: randomization? | *No* | *No* | *No* | *No* | *No* | *No* | *No* |
| 1.2 Method of patient selection or data collection: prospective? | *Yes* | *No* | *No* | *No* | *No* | *No* | *No* |
| 1.3 How representative was the patient selection in comparison with the general patients with GBMs? | *Yes* | *Yes* | *Yes* | *Yes* | *Yes* | *Yes* | *Yes* |
| *2. Comparability* |  |  |  |  |  |  |  |
| 2.1 State the distribution of important prognostic variables between the groups | *No* | *No* | *No* | *No* | *No* | *No* | *No* |
| 2.2 Group comparable for the reported variables? | *Unclear* | *Unclear* | *Unclear* | *Unclear* | *Unclear* | *Unclear* | *Unclear* |
| 2.3 Methods applied for controlling the potential prognostic confounders | *Yes* | *No* | *Yes* | *Yes* | *Yes* | *No* | *Yes* |
| ***Performance bias*** |  |  |  |  |  |  |  |
| 3. Performance |  |  |  |  |  |  |  |
| 3.1 State statistical sample size or power calculation | *No* | *No* | *No* | *No* | *No* | *No* | *No* |
| 3.2 Blinding of participants and personnel (time-to-event data)* | *Yes* | *Yes* | *Yes* | *Yes* | *Yes* | *Yes* | *Yes* |
| 3.3 Exposure of interventions | *Yes* | *Yes* | *Yes* | *Yes* | *Yes* | *Yes* | *Yes* |
| *4. Assay method* |  |  |  |  |  |  |  |
| 4.1 The method of sample handling | *Yes* | *No* | *No* | *No* | *No* | *Yes* | *No* |
| 4.2 The type of assay methods used | *Yes* | *No* | *No* | *Yes* | *No* | *Yes* | *Yes* |
| 4.3 Cutoff point determination^#^ | *Yes* | *No* | *Yes* | *Yes* | *No* | *Yes* | *Yes* |
| ***Detection bias*** |  |  |  |  |  |  |  |
| 5. Blinding of outcome assessment (time-to-event data)* | *Yes* | *Yes* | *Yes* | *Yes* | *Yes* | *Yes* | *Yes* |
| 6. Ascertainment of outcome data | *Yes* | *Yes* | *Yes* | *Yes* | *Yes* | *Yes* | *Yes* |
| ***Attrition bias*** |  |  |  |  |  |  |  |
| 7.1 Adequacy of outcome data (OS) | *Yes* | *Yes* | *Yes* | *Yes* | *Yes* | *Yes* | *Yes* |
| 7.2 Adequacy of outcome data (PFS) | *Unclear* | *Unclear* | *Unclear* | *No* | *Unclear* | *Yes* | *Unclear* |
| ***Reporting bias*** |  |  |  |  |  |  |  |
| 8. Selective outcome reporting | *Unclear* | *Unclear* | *Unclear* | *Unclear* | *Unclear* | *Unclear* | *Unclear* |

| ***Domains*** | Wick,W  2012 | *Shenouda, G.*  *2017* | Giordano, F. A  2015. | Glas, M.  2009 | Grossman, R.  2015 | Nguyen,H.N.  2017 | Brandes, A. A. 2016(recurrent GBM) |
| --- | --- | --- | --- | --- | --- | --- | --- |
| ***Selection bias*** |  |  |  |  |  |  |  |
| *1. Selection* |  |  |  |  |  |  |  |
| 1.1 Treatment assignment: randomization? | *Yes* | *No* | *No* | *No* | *No* | *No* | *Yes* |
| 1.2 Method of patient selection or data collection: prospective? | *Yes* | *Yes* | *No* | *Yes* | *No* | *No* | *Yes* |
| 1.3 How representative was the patient selection in comparison with the general patients with GBMs? | *Yes* | *Yes* | *Yes* | *Yes* | *Yes* | *Yes* | *Yes* |
| *2. Comparability* |  |  |  |  |  |  |  |
| 2.1 State the distribution of important prognostic variables between the groups | *No* | *No* | *No* | *No* | *Yes* | *No* | *No* |
| 2.2 Group comparable for the reported variables? | *Unclear* | *Unclear* | *Unclear* | *Unclear* | *Yes* | *Unclear* | *Unclear* |
| 2.3 Methods applied for controlling the potential prognostic confounders | *Yes* | *No* | *No* | *Yes* | *Yes* | *Yes* | *Yes* |
| ***Performance bias*** |  |  |  |  |  |  |  |
| 3. Performance |  |  |  |  |  |  |  |
| 3.1 State statistical sample size or power calculation | *Yes* | *Yes* | *No* | *No* | *No* | *No* | *No* |
| 3.2 Blinding of participants and personnel (time-to-event data)* | *Yes* | *Yes* | *Yes* | *Yes* | *Yes* | *Yes* | *Yes* |
| 3.3 Exposure of interventions | *Yes* | *Yes* | *Yes* | *Yes* | *Yes* | *Yes* | *Yes* |
| *4. Assay method* |  |  |  |  |  |  |  |
| 4.1 The method of sample handling | *Yes* | *No* | *No* | *No* | *Yes* | *Yes* | *No* |
| 4.2 The type of assay methods used | *Yes* | *No* | *No* | *Yes* | *Yes* | *Yes* | *Yes* |
| 4.3 Cutoff point determination^#^ | *Yes* | *No* | *No* | *Yes* | *Yes* | *Yes* | *Yes* |
| ***Detection bias*** |  |  |  |  |  |  |  |
| 5. Blinding of outcome assessment (time-to-event data)* | *Yes* | *Yes* | *Yes* | *Yes* | *Yes* | *Yes* | *Yes* |
| 6. Ascertainment of outcome data | *Yes* | *Yes* | *Yes* | *Yes* | *Yes* | *Yes* | *Yes* |
| ***Attrition bias*** |  |  |  |  |  |  |  |
| 7.1 Adequacy of outcome data (OS) | *Yes* | *Yes* | *Yes* | *Yes* | *Yes* | *Yes* | *Yes* |
| 7.2 Adequacy of outcome data (PFS) | *Yes* | *Yes* | *Yes* | *Unclear* | *Unclear* | *Yes* | *No* |
| ***Reporting bias*** |  |  |  |  |  |  |  |
| 8. Selective outcome reporting | *No* | *Unclear* | *Unclear* | *No* | *No* | *No* | *No* |

| ***Domains*** | Han, S. | Kerkhof, M. | Kim, Y. H. | *Kim, Y. S.* | *Van Mieghem, E.* | Kreth, F. W. | Weller, M. |
| --- | --- | --- | --- | --- | --- | --- | --- |
|  | 2015 | 2013 | 2015 | *2012* | *2013* | 2013 | 2015 |
| ***Selection bias*** |  |  |  |  |  |  |  |
| *1. Selection* |  |  |  |  |  |  |  |
| 1.1 Treatment assignment: randomization? | *No* | *No* | *No* | *No* | *No* | *No* | *No* |
| 1.2 Method of patient selection or data collection: prospective? | *No* | *No* | *No* | *No* | *No* | *Yes* | *Yes* |
| 1.3 How representative was the patient selection in comparison with the general patients with GBMs? | *Yes* | *Yes* | *Yes* | *Yes* | *Yes* | *Yes* | *Yes* |
| *2. Comparability* |  |  |  |  |  |  |  |
| 2.1 State the distribution of important prognostic variables between the groups | *No* | *No* | *No* | *Yes* | *Yes* | *No* | *No* |
| 2.2 Group comparable for the reported variables? | *Unclear* | *Unclear* | *Unclear* | *Yes* | *Unclear* | *Unclear* | *Unclear* |
| 2.3 Methods applied for controlling the potential prognostic confounders | *Yes* | *Yes* | *Yes* | *Yes* | *Yes* | *Yes* | *No* |
| ***Performance bias*** |  |  |  |  |  |  |  |
| 3. Performance |  |  |  |  |  |  |  |
| 3.1 State statistical sample size or power calculation | *No* | *No* | *No* | *No* | *No* | *No* | *No* |
| 3.2 Blinding of participants and personnel (time-to-event data)* | *Yes* | *Yes* | *Yes* | *Yes* | *Yes* | *Yes* | *Yes* |
| 3.3 Exposure of interventions | *Yes* | *Yes* | *Yes* | *Yes* | *Yes* | *Yes* | *Yes* |
| *4. Assay method* |  |  |  |  |  |  |  |
| 4.1 The method of sample handling | *Yes* | *No* | *No* | *Yes* | *Yes* | *No* | *No* |
| 4.2 The type of assay methods used | *Yes* | *No* | *No* | *Yes* | *Yes* | *Yes* | *Yes* |
| 4.3 Cutoff point determination^#^ | *Yes* | *No* | *No* | *Yes* | *Yes* | *Yes* | *Yes* |
| ***Detection bias*** |  |  |  |  |  |  |  |
| 5. Blinding of outcome assessment (time-to-event data)* | *Yes* | *Yes* | *Yes* | *Yes* | *Yes* | *Yes* | *Yes* |
| 6. Ascertainment of outcome data | *Yes* | *Yes* | *Yes* | *Yes* | *Yes* | *Yes* | *Yes* |
| ***Attrition bias*** |  |  |  |  |  |  |  |
| 7.1 Adequacy of outcome data (OS) | *Yes* | *Yes* | *Yes* | *Yes* | *Yes* | *Yes* | *Yes* |
| 7.2 Adequacy of outcome data (PFS) | *No* | *No* | *Unclear* | *Unclear* | *Unclear* | *Unclear* | *Yes* |
| ***Reporting bias*** |  |  |  |  |  |  |  |
| 8. Selective outcome reporting | *Unclear* | *Unclear* | *Unclear* | *Unclear* | *Unclear* | *Unclear* | *Unclear* |

| ***Domains*** | Lakomy, R. | Lee, D. | Liu, Z. | Lombardi, G. | Perry.J.R. | Metellus, P. | Metellus, P. |
| --- | --- | --- | --- | --- | --- | --- | --- |
|  | 2011 | 2013 | 2015 | 2015 | 2017 | 2009 | 2011 |
| ***Selection bias*** |  |  |  |  |  |  |  |
| *1. Selection* |  |  |  |  |  |  |  |
| 1.1 Treatment assignment: randomization? | *No* | *No* | *No* | *No* | *Yes* | *No* | *No* |
| 1.2 Method of patient selection or data collection: prospective? | *No* | *No* | *No* | *No* | *Yes* | *Yes* | *Yes* |
| 1.3 How representative was the patient selection in comparison with the general patients with GBMs? | *Yes* | *Yes* | *Yes* | *Yes* | *Yes* | *Yes* | *Yes* |
| *2. Comparability* |  |  |  |  |  |  |  |
| 2.1 State the distribution of important prognostic variables between the groups | *No* | *No* | *No* | *No* | *Yes* | *No* | *No* |
| 2.2 Group comparable for the reported variables? | *Unclear* | *Unclear* | *Unclear* | *Unclear* | *Yes* | *Unclear* | *Unclear* |
| 2.3 Methods applied for controlling the potential prognostic confounders | *No* | *Yes* | *Yes* | *Yes* | *Yes* | *Yes* | *Yes* |
| ***Performance bias*** |  |  |  |  |  |  |  |
| 3. Performance |  |  |  |  |  |  |  |
| 3.1 State statistical sample size or power calculation | *No* | *No* | *No* | *No* | *Yes* | *No* | *No* |
| 3.2 Blinding of participants and personnel (time-to-event data)* | *Yes* | *Yes* | *Yes* | *Yes* | *Yes* | *Yes* | *Yes* |
| 3.3 Exposure of interventions | *Yes* | *Yes* | *Yes* | *Yes* | *Yes* | *Yes* | *Yes* |
| *4. Assay method* |  |  |  |  |  |  |  |
| 4.1 The method of sample handling | *Yes* | *No* | *Yes* | *No* | *Yes* | *Yes* | *Yes* |
| 4.2 The type of assay methods used | *Yes* | *Yes* | *Yes* | *Yes* | *Yes* | *Yes* | *Yes* |
| 4.3 Cutoff point determination^#^ | *Yes* | *Yes* | *Yes* | *Yes* | *Yes* | *Yes* | *Yes* |
| ***Detection bias*** |  |  |  |  |  |  |  |
| 5. Blinding of outcome assessment (time-to-event data)* | *Yes* | *Yes* | *Yes* | *Yes* | *Yes* | *Yes* | *Yes* |
| 6. Ascertainment of outcome data | *Yes* | *Yes* | *Yes* | *Yes* | *Yes* | *Yes* | *Yes* |
| ***Attrition bias*** |  |  |  |  |  |  |  |
| 7.1 Adequacy of outcome data (OS) | *Yes* | *Yes* | *Yes* | *Yes* | *Yes* | *Yes* | *Yes* |
| 7.2 Adequacy of outcome data (PFS) | *Yes* | *Yes* | *Yes* | *No* | *Unclear* | *Yes* | *Yes* |
| ***Reporting bias*** |  |  |  |  |  |  |  |
| 8. Selective outcome reporting | *Unclear* | *Unclear* | *Unclear* | *Unclear* | *No* | *Unclear* | *Unclear* |

| ***Domains*** | Montano, N. | Motomura, K. | Gutenberg, A. | Murat, A. | Niyazi, M. | Park, C. K. | Rosati, A. |
| --- | --- | --- | --- | --- | --- | --- | --- |
|  | 2011 | 2011 | 2013 | 2008 | 2011 | 2009 | 2013 |
| ***Selection bias*** |  |  |  |  |  |  |  |
| *1. Selection* |  |  |  |  |  |  |  |
| 1.1 Treatment assignment: randomization? | *No* | *No* | *No* | *No* | *No* | *No* | *No* |
| 1.2 Method of patient selection or data collection: prospective? | *Yes* | *No* | *No* | *No* | *No* | *No* | *No* |
| 1.3 How representative was the patient selection in comparison with the general patients with GBMs? | *Yes* | *Yes* | *Yes* | *Yes* | *Yes* | *Yes* | *Yes* |
| *2. Comparability* |  |  |  |  |  |  |  |
| 2.1 State the distribution of important prognostic variables between the groups | *No* | *No* | *No* | *No* | *No* | *Unclear* | *No* |
| 2.2 Group comparable for the reported variables? | *Unclear* | *Unclear* | *Unclear* | *Unclear* | *Unclear* | *Unclear* | *Unclear* |
| 2.3 Methods applied for controlling the potential prognostic confounders | *Yes* | *Yes* | *No* | *Yes* | *No* | *No* | *No* |
| ***Performance bias*** |  |  |  |  |  |  |  |
| 3. Performance |  |  |  |  |  |  |  |
| 3.1 State statistical sample size or power calculation | *No* | *No* | *No* | *No* | *No* | *No* | *No* |
| 3.2 Blinding of participants and personnel (time-to-event data)* | *Yes* | *Yes* | *Yes* | *Yes* | *Yes* | *Yes* | *Yes* |
| 3.3 Exposure of interventions | *Yes* | *Yes* | *Yes* | *Yes* | *Yes* | *Yes* | *Yes* |
| *4. Assay method* |  |  |  |  |  |  |  |
| 4.1 The method of sample handling | *Yes* | *Yes* | *Yes* | *Yes* | *No* | *Yes* | *Yes* |
| 4.2 The type of assay methods used | *Yes* | *Yes* | *Yes* | *Unclear* | *Yes* | *Yes* | *Yes* |
| 4.3 Cutoff point determination^#^ | *Yes* | *Yes* | *Yes* | *Unclear* | *Yes* | *Yes* | *Yes* |
| ***Detection bias*** |  |  |  |  |  |  |  |
| 5. Blinding of outcome assessment (time-to-event data)* | *Yes* | *Yes* | *Yes* | *Yes* | *Yes* | *Yes* | *Yes* |
| 6. Ascertainment of outcome data | *Yes* | *Yes* | *Yes* | *Yes* | *Yes* | *Yes* | *Yes* |
| ***Attrition bias*** |  |  |  |  |  |  |  |
| 7.1 Adequacy of outcome data (OS) | *Yes* | *Yes* | *Yes* | *Yes* | *Yes* | *Yes* | *Yes* |
| 7.2 Adequacy of outcome data (PFS) | *No* | *No* | *Yes* | *No* | *No* | *No* | *No* |
| ***Reporting bias*** |  |  |  |  |  |  |  |
| 8. Selective outcome reporting | *Unclear* | *Unclear* | *Unclear* | *Unclear* | *Unclear* | *Unclear* | *Unclear* |

| ***Domains*** | Sana, J.  2014 | Saraiva-Espe  ron, U.(A) 2014 | Schaich, M.  2009 | Lombardi, G.  2016 | Minniti,G.  2015 | Soffietti, R.  2014 | Minniti, G.  2011(recurrent GBM) |
| --- | --- | --- | --- | --- | --- | --- | --- |
| ***Selection bias*** |  |  |  |  |  |  |  |
| *1. Selection* |  |  |  |  |  |  |  |
| 1.1 Treatment assignment: randomization? | *No* | *No* | *No* | *No* | *No* | *No* | *No* |
| 1.2 Method of patient selection or data collection: prospective? | *No* | *No* | *No* | *No* | *No* | *Yes* | *Yes* |
| 1.3 How representative was the patient selection in comparison with the general patients with GBMs? | *Yes* | *Yes* | *Yes* | *Yes* | *Yes* | *Yes* | *Yes* |
| *2. Comparability* |  |  |  |  |  |  |  |
| 2.1 State the distribution of important prognostic variables between the groups | *No* | *No* | *No* | *No* | *No* | *No* | *No* |
| 2.2 Group comparable for the reported variables? | *Unclear* | *Unclear* | *Unclear* | *Unclear* | *Unclear* | *Unclear* | *Unclear* |
| 2.3 Methods applied for controlling the potential prognostic confounders | *No* | *Yes* | *Yes* | *No* | *Yes* | *Yes* | *Yes* |
| ***Performance bias*** |  |  |  |  |  |  |  |
| 3. Performance |  |  |  |  |  |  |  |
| 3.1 State statistical sample size or power calculation | *No* | *No* | *No* | *No* | *No* | *No* | *No* |
| 3.2 Blinding of participants and personnel (time-to-event data)* | *Yes* | *Yes* | *Yes* | *Yes* | *Yes* | *Yes* | *Yes* |
| 3.3 Exposure of interventions | *Yes* | *Yes* | *Yes* | *Yes* | *Yes* | *Yes* | *Yes* |
| *4. Assay method* |  |  |  |  |  |  |  |
| 4.1 The method of sample handling | *Yes* | *Yes* | *Yes* | *No* | *No* | *Yes* | *No* |
| 4.2 The type of assay methods used | *Yes* | *Yes* | *Yes* | *Yes* | *Yes* | *Yes* | *Yes* |
| 4.3 Cutoff point determination^#^ | *Yes* | *Yes* | *Yes* | *No* | *Yes* | *Yes* | *Yes* |
| ***Detection bias*** |  |  |  |  |  |  |  |
| 5. Blinding of outcome assessment (time-to-event data)* | *Yes* | *Yes* | *Yes* | *Yes* | *Yes* | *Yes* | *Yes* |
| 6. Ascertainment of outcome data | *Yes* | *Yes* | *Yes* | *Yes* | *Yes* | *Yes* | *Yes* |
| ***Attrition bias*** |  |  |  |  |  |  |  |
| 7.1 Adequacy of outcome data (OS) | *Yes* | *Yes* | *Yes* | *Yes* | *Yes* | *Yes* | *Yes* |
| 7.2 Adequacy of outcome data (PFS) | *Yes* | *No* | *No* | *Yes* | *Yes* | *Yes* | *Yes* |
| ***Reporting bias*** |  |  |  |  |  |  |  |
| 8. Selective outcome reporting | *Unclear* | *Unclear* | *Unclear* | *Unclear* | *Unclear* | *No* | *Unclear* |

| ***Domains*** | Zhang, X. Q.  2013 | Brandes, A. A.  2016(GBM) | Jungk, C  2016. | Yang, P  2015. | *Yang, M.*  *2013* | Ohno, M.  2016 | Stummer, W.  2012 |  |
| --- | --- | --- | --- | --- | --- | --- | --- | --- |
| ***Selection bias*** |  |  |  |  |  |  |  |  |
| *1. Selection* |  |  |  |  |  |  |  |  |
| 1.1 Treatment assignment: randomization? | *No* | *No* | *No* | *No* | *No* | *No* | *No* |  |
| 1.2 Method of patient selection or data collection: prospective? | *No* | *Yes* | *No* | *Yes* | *No* | *Yes* | *Yes* |  |
| 1.3 How representative was the patient selection in comparison with the general patients with GBMs? | *Yes* | *Yes* | *Yes* | *Yes* | *Yes* | *Yes* | *Yes* |  |
| *2. Comparability* |  |  |  |  |  |  |  |  |
| 2.1 State the distribution of important prognostic variables between the groups | *No* | *No* | *No* | *No* | *No* | *No* | *No* |  |
| 2.2 Group comparable for the reported variables? | *Unclear* | *Unclear* | *Unclear* | *Unclear* | *Unclear* | *Unclear* | *Unclear* |  |
| 2.3 Methods applied for controlling the potential prognostic confounders | *Yes* | *Yes* | *Yes* | *Yes* | *Yes* | *Yes* | *No* |  |
| ***Performance bias*** |  |  |  |  |  |  |  |  |
| 3. Performance |  |  |  |  |  |  |  |  |
| 3.1 State statistical sample size or power calculation | *No* | *No* | *No* | *No* | *No* | *No* | *No* |  |
| 3.2 Blinding of participants and personnel (time-to-event data)* | *Yes* | *Yes* | *Yes* | *Yes* | *Yes* | *Yes* | *Yes* |  |
| 3.3 Exposure of interventions | *Yes* | *Yes* | *Yes* | *Yes* | *Yes* | *Yes* | *Yes* |  |
| *4. Assay method* |  |  |  |  |  |  |  |  |
| 4.1 The method of sample handling | *No* | *Yes* | *No* | *Yes* | *Yes* | *Yes* | *Yes* |  |
| 4.2 The type of assay methods used | *No* | *Yes* | *Yes* | *Yes* | *Yes* | *Yes* | *Yes* |  |
| 4.3 Cutoff point determination^#^ | *No* | *Yes* | *Yes* | *Yes* | *Yes* | *Yes* | *Yes* |  |
| ***Detection bias*** |  |  |  |  |  |  |  |  |
| 5. Blinding of outcome assessment (time-to-event data)* | *Yes* | *Yes* | *Yes* | *Yes* | *Yes* | *Yes* | *Yes* |  |
| 6. Ascertainment of outcome data | *Yes* | *Yes* | *Yes* | *Yes* | *Yes* | *Yes* | *Yes* |  |
| ***Attrition bias*** |  |  |  |  |  |  |  |  |
| 7.1 Adequacy of outcome data (OS) | *Yes* | *Yes* | *Yes* | *Yes* | *Yes* | *Unclear* | *Yes* |  |
| 7.2 Adequacy of outcome data (PFS) | *No* | *No* | *Unclear* | *No* | *No* | *Yes* | *No* |  |
| ***Reporting bias*** |  |  |  |  |  |  |  |  |
| 8. Selective outcome reporting | *Unclear* | *Unclear* | *Unclear* | *Unclear* | *Unclear* | *Unclear* | *Unclear* |  |

| ***Domains*** | Ma, C. | Kim, C. | Schaub, C. | Thon, N. | Vaios, E. J. | Malmstrom, A | Clarke, J. L. |
| --- | --- | --- | --- | --- | --- | --- | --- |
|  | 2016 | 2017 | 2016 | 2017 | 2016 | 2012 | 2009 |
| ***Selection bias*** |  |  |  |  |  |  |  |
| *1. Selection* |  |  |  |  |  |  |  |
| 1.1 Treatment assignment: randomization? | *No* | *No* | *No* | *No* | *No* | *Yes* | *Yes* |
| 1.2 Method of patient selection or data collection: prospective? | *No* | *No* | *No* | *Yes* | *No* | *Yes* | *Yes* |
| 1.3 How representative was the patient selection in comparison with the general patients with GBMs? | *Yes* | *Yes* | *Yes* | *Yes* | *Yes* | *Yes* | *Yes* |
| *2. Comparability* |  |  |  |  |  |  |  |
| 2.1 State the distribution of important prognostic variables between the groups | *No* | *No* | *No* | *Yes* | *No* | *No* | *No* |
| 2.2 Group comparable for the reported variables? | *Unclear* | *Unclear* | *Unclear* | *Yes* | *Unclear* | *Unclear* | *Unclear* |
| 2.3 Methods applied for controlling the potential prognostic confounders | *Yes* | *Yes* | *No* | *Yes* | *Yes* | *Yes* | *Yes* |
| ***Performance bias*** |  |  |  |  |  |  |  |
| 3. Performance |  |  |  |  |  |  |  |
| 3.1 State statistical sample size or power calculation | *No* | *No* | *No* | *Yes* | *No* | *Yes* | *Yes* |
| 3.2 Blinding of participants and personnel (time-to-event data)* | *Yes* | *Yes* | *Yes* | *Yes* | *Yes* | *Yes* | *Yes* |
| 3.3 Exposure of interventions | *Yes* | *Yes* | *Yes* | *Yes* | *Yes* | *Yes* | *Yes* |
| *4. Assay method* |  |  |  |  |  |  |  |
| 4.1 The method of sample handling | *No* | *Yes* | *No* | *No* | *No* | *Yes* | *Yes* |
| 4.2 The type of assay methods used | *Yes* | *Yes* | *No* | *Yes* | *No* | *Yes* | *Yes* |
| 4.3 Cutoff point determination^#^ | *Yes* | *Yes* | *No* | *Yes* | *No* | *Yes* | *unclear* |
| ***Detection bias*** |  |  |  |  |  |  |  |
| 5. Blinding of outcome assessment (time-to-event data)* | *Yes* | *Yes* | *Yes* | *Yes* | *Yes* | *Yes* | *Yes* |
| 6. Ascertainment of outcome data | *Yes* | *Yes* | *Yes* | *Yes* | *Yes* | *Yes* | *Yes* |
| ***Attrition bias*** |  |  |  |  |  |  |  |
| 7.1 Adequacy of outcome data (OS) | *Yes* | *Unclear* | *Yes* | *Yes* | *Yes* | *Yes* | *Yes* |
| 7.2 Adequacy of outcome data (PFS) | *Unclear* | *Yes* | *No* | *Yes* | *No* | *Unclear* | *Unclear* |
| ***Reporting bias*** |  |  |  |  |  |  |  |
| 8. Selective outcome reporting | *Unclear* | *Unclear* | *Unclear* | *Unclear* | *Unclear* | *No* | *No* |

| Domains | Gallego Perez-Larraya, J.  2011 | Lai, A.  2011 | Lam, N.  2012 | Stupp, R.  2010 | Gilbert,M.R.  2013 | Saraiva-Espe  ron, U.(B) 2014 | Arita, H.  2016 |
| --- | --- | --- | --- | --- | --- | --- | --- |
| ***Selection bias*** |  |  |  |  |  |  |  |
| *1. Selection* |  |  |  |  |  |  |  |
| 1.1 Treatment assignment: randomization? | *No* | *No* | *No* | *No* | *Yes* | *No* | *No* |
| 1.2 Method of patient selection or data collection: prospective? | *Yes* | *Yes* | *No* | *Yes* | *Yes* | *No* | *No* |
| 1.3 How representative was the patient selection in comparison with the general patients with GBMs? | *Yes* | *Yes* | *Yes* | *Yes* | *Yes* | *Yes* | *Yes* |
| *2. Comparability* |  |  |  |  |  |  |  |
| 2.1 State the distribution of important prognostic variables between the groups | *No* | *No* | *No* | *No* | *No* | *No* | *No* |
| 2.2 Group comparable for the reported variables? | *Unclear* | *Unclear* | *Unclear* | *Unclear* | *Unclear* | *Unclear* | *Unclear* |
| 2.3 Methods applied for controlling the potential prognostic confounders | *Yes* | *Yes* | *No* | *No* | *No* | *Yes* | *Yes* |
| ***Performance bias*** |  |  |  |  |  |  |  |
| 3. Performance |  |  |  |  |  |  |  |
| 3.1 State statistical sample size or power calculation | *Yes* | *Yes* | *No* | *Yes* | *Yes* | *No* | *No* |
| 3.2 Blinding of participants and personnel (time-to-event data)* | *Yes* | *Yes* | *Yes* | *Yes* | *Yes* | *Yes* | *Yes* |
| 3.3 Exposure of interventions | *Yes* | *Yes* | *Yes* | *Yes* | *Yes* | *Yes* | *Yes* |
| *4. Assay method* |  |  |  |  |  |  |  |
| 4.1 The method of sample handling | *Yes* | *Yes* | *No* | *Yes* | *No* | *Unclear* | *Yes* |
| 4.2 The type of assay methods used | *Yes* | *Yes* | *Yes* | *Yes* | *Yes* | *Yes* | *Yes* |
| 4.3 Cutoff point determination^#^ | *Yes* | *Yes* | *Unclear* | *Yes* | *Yes* | *Unclear* | *Yes* |
| ***Detection bias*** |  |  |  |  |  |  |  |
| 5. Blinding of outcome assessment (time-to-event data)* | *Yes* | *Yes* | *Yes* | *Yes* | *Yes* | *Yes* | *Yes* |
| 6. Ascertainment of outcome data | *Yes* | *Yes* | *Yes* | *Yes* | *Yes* | *Yes* | *Yes* |
| ***Attrition bias*** |  |  |  |  |  |  |  |
| 7.1 Adequacy of outcome data (OS) | *Yes* | *Yes* | *Yes* | *Yes* | *Yes* | *Yes* | *Yes* |
| 7.2 Adequacy of outcome data (PFS) | *Unclear* | *Yes* | *Unclear* | *Yes* | *Yes* | *Unclear* | *Yes* |
| ***Reporting bias*** |  |  |  |  |  |  |  |
| 8. Selective outcome reporting | *No* | *No* | *Unclear* | *No* | *No* | *Unclear* | *Unclear* |

## * Yes was assigned despite that blinding was not conducted because the reviewers authors judged that OS and PFS were unlikely to be influenced by lack of blinding.

# Yes was assigned if the assay methods applied had qualitativelybinary readouts.

**Supplementary Table S2. Criteria for judgment of risk of bias in the modified domain-based Newcastle-Ottawa Scale (NOS)**

**(**[**Higgins and Green, 2011**](#_ENREF_2)**;** [**Wells et al., 2011**](#_ENREF_3)**;** [**Altman et al., 2012**](#_ENREF_1)**)**

| ***Domains*** | **Judgment criteria for responses to each domain** | | |
| --- | --- | --- | --- |
|  | ***Yes*** | ***No*** | ***Unclear*** |
| ***Selection bias*** |  |  |  |
| *Selection* |  |  |  |
| Treatment assignment: randomization? | *The investigators describe a random component in the sequence generation process such as coin tossing, using a computer random number generator, etc.* | *The investigators describe a non-random component in the sequence generation process such as sequence generated based on hospital or clinical record number, allocation by*  *judgment of the clinician, etc.* | *Insufficient description* |
| How representative was the patient selection in comparison with the general | *Truly or somewhat representative of the general population with GBMs* | *Selected group of e.g. doctors, nurses* | *Insufficient description* |
| *Comparability* |  |  |  |
| State the distribution of the following important prognostic variables (age, gender, KPS or ECOG performance score, surgery, adjuvant treatment, co-morbidity, tumor location or numbers; neurological status；primary or recurrent GBM) between the  groups with different MGMT statues | *Most of the variables (e.g., at least 4 out of 8 items) were reported* | *At least two variables were not reported* | *Not applicable* |
| Groups comparable for the above mentioned  variables | *All reported variables were comparable*  *between the groups* | *At least one of those was not comparable* | *Comparability was not applicable*  *because insufficient data were reported* |
| Methods applied for controlling the potential prognostic confounders | *Appropriate methods are used to control the potential confounders (e.g. multivariate*  *modeling, matching, etc)* | *No method was applied to control the potential confounders* | *Insufficient description* |
| ***Performance bias*** |  |  |  |
| *Performance* |  |  |  |
| State statistical sample size or power  calculation | *Reported* | *Not reported* | *Not applicable* |
| Blinding of participants and personnel | 1. *No blinding or incomplete blinding, but the reviewers judge that the outcome is not likely to be influenced by lack ofblinding* 2. *Blinding of key study participants and personnel and unlikely that the blinding could beenbroken* | 1. *No blinding or incomplete blinding, the outcome is likely to be influenced by lack of blinding* 2. *Blinding of key study participants and personnel and likely that the blindingcould*   *been broken, and the outcome is likely to be influenced by lack of blinding* | *Insufficient description or the study did not address the outcome* |

| Ascertainment of intervention exposure | *Medical records or structured interview* | *Written self report* | *Insufficient description* |  |
| --- | --- | --- | --- | --- |
| *Assay method* |  |  |  |  |
| The method of sample handling | *Reported* | *Not reported* | *Not applicable* |  |
| The type of assay methods used | *Reported* | *Not reported* | *Not applicable* |  |
| Cutoff point determination | *Reported* | *Not reported* | *Not applicable* |  |
| ***Detection bias*** |  |  |  |  |
| Blinding of outcome assessment | 1. *No blinding of outcome assessment, but the reviewers judge that the outcome measurement is not likely to be influenced by lack of blinding.* 2. *Blinding of outcome assessment ensured, and unlikely that the blinding could have been broken.* | 1. *No blinding of outcome assessment, the outcome measurement is likely to be influenced by lack of blinding.* 2. *Blinding of outcome assessment ensured, and likely that the blinding could have been broken and the outcome assessment is likely to be influenced by lack of blinding.* | *Insufficient description or the study did not address the outcome* |  |
| Ascertainment of outcome data | *Record linkage* | *Self report* | *Insufficient description* |  |
| ***Attrition bias*** |  |  |  |  |
| Adequacy of outcome data | 1. *The follow-up was long enough for outcomes tooccur* 2. *Adequate follow up (e.g.≥80%) or subjects lost to follow up unlikely to introduce bias (e.g. for survival data, censoring unlikely to be introducing bias)* | 1. *The follow-up was not long enough for outcomes tooccur* 2. *Inadequate follow up (e.g.<80%)or subjects lost to follow up are very likely to introduce bias (e.g. for dichotomousoutcome data, the proportion of missingoutcomes*   *compared with observed event risk enough to induce bias)* | *Insufficient description or the study did not address the outcome* |  |
| ***Reporting bias*** |  |  |  |  |
| Selective outcome reporting | 1. *the study protocol is available and all of the study’s pre-specified outcomes of interest in the review have been reported inthe*   *pre-specified way*   1. *the study protocol is not available but itis clear that the published reports included all expectedoutcomes* | 1. *not all of pre-specified outcomes have been reported* 2. *one or more outcomes were reported in a way that were notpre-specified* 3. *one or more outcomes were reported incompletely so that they cannot be entered in ameta-analysis* 4. *the study report failed to include resultsfor*   *a key outcome that would be expected to have been reported for such a study* | *Insufficient description** |  |

RT=radiotherapy; TMZ=temozolomide; GBM: glioblastoma; KPS= Karnofsky performance status; MGMT=O6-methylguanine-DNA methyltransferase

*It is very likely that the majority of studies will fall into this category, especially for those that the study protocol is not available.

**References:**

Altman, D.G., McShane, L.M., Sauerbrei, W., and Taube, S.E. (2012). Reporting recommendations for tumor marker prognostic studies (REMARK): explanation and elaboration. *BMC medicine* 10(1)**,** 51.

Higgins, J.P., and Green, S. (2011). *Cochrane handbook for systematic reviews of interventions.* John Wiley & Sons.

Wells, G., Shea, B., O’Connell, D., Peterson, J., Welch, V., Losos, M., et al. (2011). "The Newcastle-Ottawa scale (NOS) for assessing the quality of nonrandomized studies in meta-analysis. Ottawa, Ontario: The Ottawa Health Research Institute".).

**Supplement table S3: Summary of subgroup analysis with multivariate studies**

| **Variable** | **Subgroup** | **Treatment** | **Trial**  **(N)** | **HR**  **(95% CI)** | **P value**  **for HR** | **Bon** | ***I^2^*** | **P value**  **(Egger's)** |
| --- | --- | --- | --- | --- | --- | --- | --- | --- |

|  | OS analysis (methylated vs un-methylated) | | | | | | | | | |
| --- | --- | --- | --- | --- | --- | --- | --- | --- | --- | --- |
| Overall | | |  | TMZ-containing | 34 | 0.43(0.37-0.51) | <0.001 | 0.012 | 74.8% | 0.015 |
|  | | |  | TMZ-free | 6 | 0.79(0.66-0.95) | 0.013 | 0.143 | 0% | NA |
| Race | | Caucasian | | TMZ-containing | 19 | 0.41(0.31-0.54) | <0.001 | 0.012 | 81.2% | 0.003 |
|  | |  | | TMZ-free | 2 | 0.87(0.55-1.35) | 0.52 | 3.64 | 0% | NA |
|  | | Asian | | TMZ-containing | 10 | 0.48(0.42-0.54) | <0.001 | 0.012 | 43.8% | 0.26 |
|  | |  | | TMZ-free | 4 | 0.78(0.64-0.95) | 0.015 | 0.135 | 0% | NA |
|  | | Mixed race | | TMZ-containing | 5 | 0.45(0.30-0.67) | <0.001 | 0.012 | 78.8% | NA |
|  | |  | | TMZ- free | 0 | NA | NA | NA | NA | NA |
| GBM Type | | Newly diagnosed | | TMZ-containing | 32 | 0.44(0.37-0.52) | <0.001 | 0.012 | 75.9% | 0.024 |
|  | |  | | TMZ-free | 3 | 0.75(0.59-0.94) | 0.014 | 0.14 | 0% | NA |
|  | | Elderly | | TMZ-containing | 4 | 0.32(0.24-0.41) | <0.001 | 0.012 | 0% | NA |
|  | |  | | TMZ-free | 0 | NA | NA | NA | NA | NA |
|  | | Recurrent | | TMZ-containing | 2 | 0.32(0.16-0.64) | 0.001 | 0.012 | 0% | NA |
|  | |  | | TMZ-free | 3 | 087(0.65-1.17) | 0.36 | 2.88 | 0% | NA |
|  | PFS analysis(methylated vs un-methylated) | | | | | | | | | |
| Overall | |  | | TMZ-containing | 12 | 0.41(0.35-0.49) | <0.001 | 0.01 | 40.4% | 0.22 |
|  |  |  | | TMZ-free | 2 | 0.79(0.66-0.95) | 0.005 | 0.045 | 0% | NA |
| Race | | Caucasian | | TMZ-containing | 5 | 0.30(0.24-0.38) | <0.001 | 0.01 | 0% | NA |
|  | |  | | TMZ-free | 1 | 0.48(0.21-1.09) | 0.08 | 0.56 | NA | NA |
|  | | Asian | | TMZ-containing | 5 | 0.49(0.41-0.59) | <0.001 | 0.01 | 0% | NA |
|  | |  | | TMZ-free | 1 | 0.69(0.50-0.94) | 0.02 | 0.16 | NA | NA |
|  |  | Mixed race | | TMZ-containing | 2 | 0.44(0.35-0.55) | <0.001 | 0.01 | NA | NA |
|  |  |  | | TMZ-free | 0 | NA | NA | NA | NA | NA |
| GBM Type | | Newly diagnosed | | TMZ-containing | 9 | 0.42(0.36-0.51) | <0.001 | 0.01 | 38.5% | 0.44 |
|  | |  | | TMZ-free | 0 | NA | NA | NA | NA | NA |
|  | | Elderly | | TMZ-containing | 0 | NA | NA | NA | NA | NA |
|  | |  | | TMZ-free | 0 | NA | NA | NA | NA | NA |
|  | | Recurrent | | TMZ-containing | 3 | 0.32(0.17-0.60) | <0.001 | 0.01 | 56.2% | NA |
|  | |  | | TMZ-free | 2 | 0.66(0.49-0.88) | 0.005 | 0.045 | 0% | NA |

HR: hazard ratio; CI: confidence interval; NA: not applicable; TMZ-containing treatment: TMZ-alone and combined radiotherapy/TMZ and combined radiotherapy/TMZ-containing chemotherapy; TMZ-free treatment: radiotherapy alone and combined radiotherapy/TMZ-free alkylation agents chemotherapy; Mixed race: patients in American studies. ; Bon: P for Step-down Bonferroni adjustment.
